# Freezing-induced wetting transitions on superhydrophobic surfaces

**DOI:** 10.1038/s41567-023-01946-3

**Published:** 2023-02-09

**Authors:** Henry Lambley, Gustav Graeber, Raphael Vogt, Leon C. Gaugler, Enea Baumann, Thomas M. Schutzius, Dimos Poulikakos

**Affiliations:** 1 Laboratory of Thermodynamics in Emerging Technologies, Department of Mechanical and Process Engineering, ETH Zurich, Zurich, Switzerland; 2 Laboratory for Multiphase Thermofluidics and Surface Nanoengineering, Department of Mechanical and Process Engineering, ETH Zurich, Zurich, Switzerland

**Keywords:** Applied physics, Fluid dynamics

## Abstract

Supercooled droplet freezing on surfaces occurs frequently in nature and industry, often adversely affecting the efficiency and reliability of technological processes. The ability of superhydrophobic surfaces to rapidly shed water and reduce ice adhesion make them promising candidates for resistance to icing. However, the effect of supercooled droplet freezing—with its inherent rapid local heating and explosive vaporization—on the evolution of droplet–substrate interactions, and the resulting implications for the design of icephobic surfaces, are little explored. Here we investigate the freezing of supercooled droplets resting on engineered textured surfaces. On the basis of investigations in which freezing is induced by evacuation of the atmosphere, we determine the surface properties required to promote ice self-expulsion and, simultaneously, identify two mechanisms through which repellency falters. We elucidate these outcomes by balancing (anti-)wetting surface forces with those triggered by recalescent freezing phenomena and demonstrate rationally designed textures to promote ice expulsion. Finally, we consider the complementary case of freezing at atmospheric pressure and subzero temperature, where we observe bottom-up ice suffusion within the surface texture. We then assemble a rational framework for the phenomenology of ice adhesion of supercooled droplets throughout freezing, informing ice-repellent surface design across the phase diagram.

## Abstract (web summary)

Icephobic surfaces are helpful for increasing safety and sustainability in engineering applications. A study of the behaviour of supercooled droplets freezing on superhydrophobic surfaces now provides insights into ice-repellency mechanisms.

## Main

The freezing of droplets on surfaces is commonplace in nature and holds relevance for the efficacy and safety of transportation, construction and power generation^1,2^. Current ice-repellency approaches are resource intensive, relying on the use of chemicals or high energy consumption^3^. However, sustainability considerations have fuelled the need for icephobic surfaces, which passively prevent the accretion of ice through shedding supercooled droplets^4–6^, delaying the onset of freezing^7–11^ or having low adhesion to already-formed ice^12–18^. Interestingly, the often-overlooked physics of droplet freezing, a two-stage process of rapid, quasi-adiabatic recalescence followed by slower crystallization^19,20^, has recently revealed intriguing physical phenomena that might be utilized to further enhance icephobicity. The latent heat released during recalescence can cause explosive vaporization leading to levitation^21^, frost halo formation^22^ and cascade freezing^23^, while volumetric expansion during crystallization can lead to droplet self-peeling^24^ and disintegration^25^. Despite progress in understanding condensation frosting and droplet freezing on hydrophobic surfaces, little research has investigated or exploited related emerging phenomena resulting from the non-equilibrium freezing of water from a supercooled state. Here we explore the freezing behaviour of supercooled droplets on superhydrophobic surfaces across a range of ambient temperatures and pressures. At low pressure, we establish the substrate characteristics necessary to induce ice-expulsion behaviour—a unique phenomenon realizing passive anti-icing—that is driven by the explosive latent heat release during freezing. Concurrently, we identify two distinct mechanisms through which surface ice repellency breaks down. We also provide experimental evidence, a theoretical model and effective design solutions to rationalize and mitigate these effects. Finally, at atmospheric pressure and reduced temperature, we discover a contrasting set of phenomena leading to bottom-up ice suffusion within the superhydrophobic surface texture. We expect that this work will provide a blueprint for the design of robust icephobic surfaces—which are needed in applications such as aviation, civil infrastructure and power transmission—across a wide range of potential operating conditions.

To systematically investigate the dynamics of water droplets freezing on superhydrophobic surfaces, we fabricated regular arrays of transparent cylindrical micropillar textures from polydimethylsiloxane (PDMS) using soft lithography. Synchronous side- and bottom-view observations were obtained using a high-speed camera and an inverted interferometer, respectively. In an environmental chamber at ambient temperature (*T*_∞_ = 22 ± 1 °C; mean and standard deviation of 249 experiments), freezing was initiated by exposing water droplets with initial volume *V* = 10 μl (if not stated otherwise) in the Cassie–Baxter^26^ wetting state to a dry, low-pressure environment (ambient pressure, *P* ≈ 0.1 kPa and relative humidity, RH ≈ 0%, see ‘Relative humidity’ in the Supplementary Information for the effect of RH) in which they rapidly supercooled through evaporation to a nucleation temperature of ≈−15 °C with an experimental uncertainty of ± 3 °C. Owing to the cooling method employed, nucleation at the free surface was favourable^20^. Supercooled water droplets freeze in two stages: first, recalescence, a kinetic-limited quasi-adiabatic process during which the freezing front propagates rapidly across the supercooled droplet (∼10 ms), increasing the temperature to its theoretical equilibrium value and resulting in an opaque liquid–solid slush, followed by the much slower stage towards complete crystallization (∼1 s) in which the remaining liquid solidifies at the solid–liquid equilibrium temperature^19,20^. Previous research has largely neglected the impact of explosive vaporization due to recalescence freezing across a broad range of environmental and surface conditions, and how it affects the resultant frozen droplet–substrate interaction. As these intrinsic phenomena can have a dominant impact on the icephobic performance of a superhydrophobic surface, their elucidation constitutes the focus of this study.

**Fig. 1:**
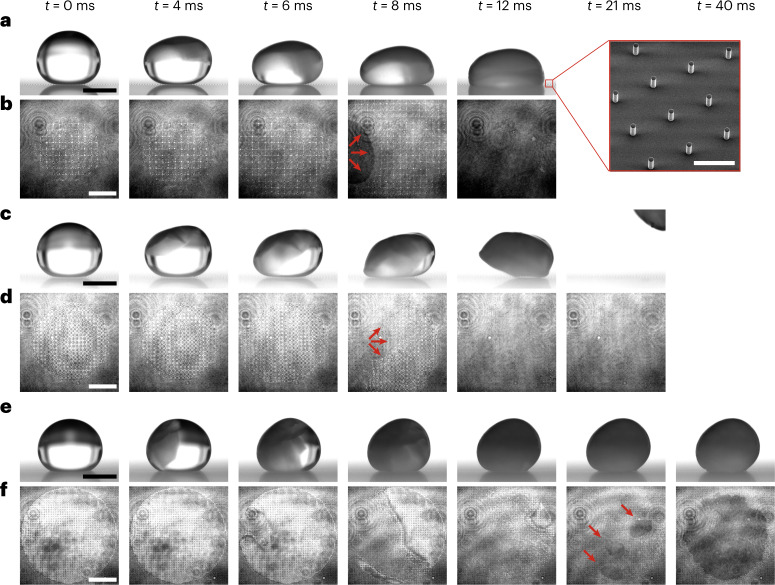
Freezing-induced droplet dynamics on superhydrophobic surfaces. **a**–**f**, Synchronized side- (**a**,**c**,**e**) and bottom- (**b**,**d**,**f**) view image sequences of water droplets freezing through evaporative cooling in a dry, low-pressure environment with different outcomes. **a**,**b**, Impalement: penetration of the meniscus into the texture characterized by a low final contact angle and full substrate wetting (the dark area in **b**; red arrows illustrate the spreading direction of the penetrated liquid). The inset in **a** is a micrograph of the transparent superhydrophobic micropillar surface (scale bar, 100 μm). **c**,**d**, Expulsion: spontaneous de-wetting of the droplet (the receding contact line is marked by red arrows). **e**,**f**, Suffusion: freezing on top of the texture characterized by a high final contact angle, followed by volumetric expansion into the texture (the dark area in **f**; red arrows indicate the initial regions of substrate wetting). Scale bars: **a**,**c**,**e**, 2 mm; **b**,**d**,**f**, 500 μm. Employed surfaces: **a**,**b**, D6; **c**,**d**, D4; **e**,**f**, D1 (see Supplementary Table 1 for details).

Figure 1 highlights progressions of the recalescent freezing of supercooled water droplets deposited on superhydrophobic PDMS micropillar surfaces viewed from the side (Fig. 1a,c,e) and below (Fig. 1b,d,f). Each sequence shown initiates in a similar manner with nucleation from the free surface and the droplet self-deforming substantially from that point, but they result in three markedly different outcomes. In the first (Fig. 1a,b and Supplementary Video 1), which we shall refer to as impalement, the droplet compresses itself downwards with the contact line spreading across the texture before liquid penetrates between the micropillars beneath the droplet and wets the bottom of the texture, completing a transition from Cassie–Baxter to Wenzel wetting^26–31^. The final state is characterized by a reduced macroscopic contact angle well below the initial value of ~150° from the side view and the extinguishing of interference fringes (bright white areas between pillars) and darkening of the bottom view owing to the removal of the liquid–air and solid–air interfaces beneath the droplet^32–34^. Fig. 1c,d depicts expulsion behaviour in which the contact line recedes as the droplet lifts itself away from the surface, leaving a clean substrate (Supplementary Video 2). Any lateral velocity imparted on the droplet reflects the ice nucleation position being away from the droplet zenith. In the final observed outcome of suffusion (Fig. 1e,f and Supplementary Video 3), the droplet remains stationary over the course of recalescence and retains the high initial contact angle of the liquid phase. From below, the droplet seems to disappear owing to scattering at the droplet–air interface from the ice–water slush formed during recalescence. However, as crystallization begins, wetting of the texture cavities is observed, starting from one or more discrete points before spreading to an area similar to that of the initial footprint of the droplet (Supplementary Fig. 1).

**Fig. 2:**
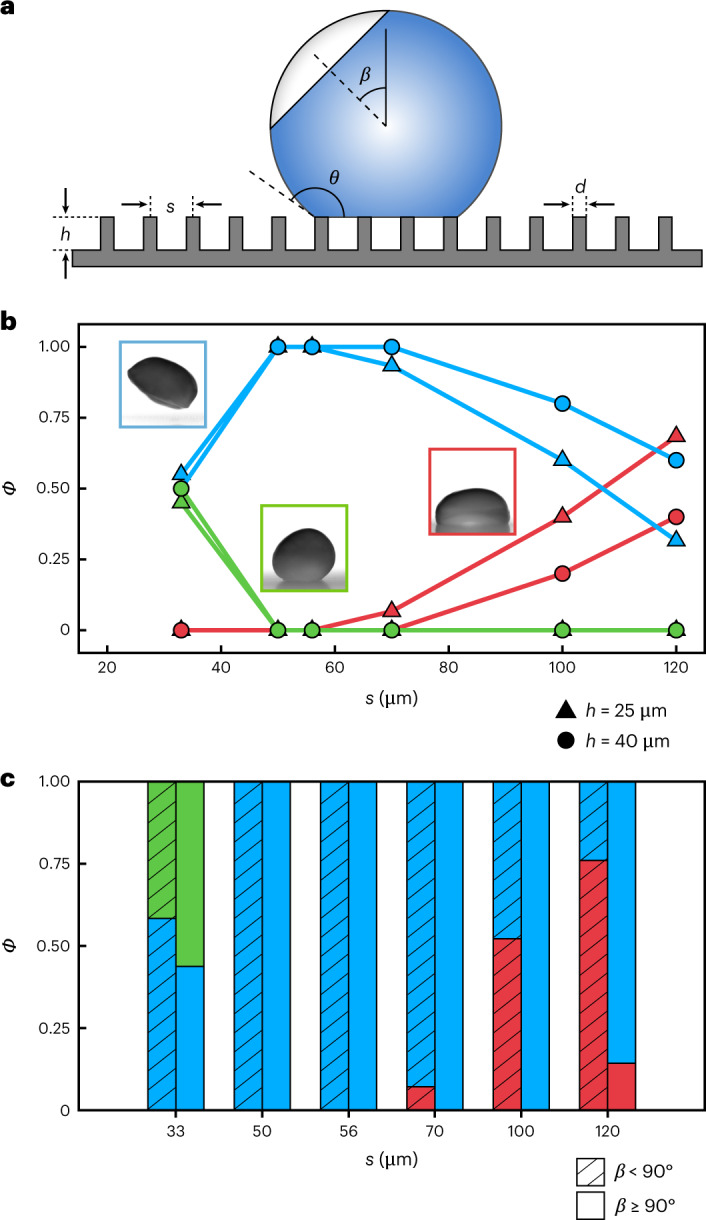
Microtexture topography and freezing characteristics alter freezing outcomes. **a**, Schematic of a droplet resting on a superhydrophobic surface (not to scale) during recalescence, introducing *β*, s, d, h and the contact angle (*θ*). In the schematic, blue represents supercooled water and the grey area shows the progression of the freezing front. **b**, *Φ* versus *s* for water droplets in a low-pressure environment. Outcomes are differentiated by colour (red, impalement; blue, expulsion; green, suffusion) for two pillar heights (*h* = 25 µm and 40 µm). **c**, Bar chart of *Φ* for each *s* as a function of *β* (*N* = 249, *n* ≥ 19 experiments per data point). Employed surfaces: D1 to D6 (see Supplementary Table 1 for details). Source data

To systematically study the effect of the surface texture on the freezing outcome, we produced samples with varying interpillar pitches of *s* = 33, 50, 56, 70, 100 and 120 µm and heights *h* = 25 and 40 µm while maintaining constant diameters of *d* = 10 µm (Supplementary Table 1). Another variable that we recorded but could not control (owing to the stochastic nature of phase change) is the angle at which nucleation initiates with respect to the droplet zenith, *β* (Fig. 2a). Note that owing to the initially (before nucleation) axisymmetric nature of the problem, we can neglect the effect of the azimuthal nucleation angle. The outcome probabilities, *Φ*, for all experiments performed (*N* = 249) for the 12 different micropillar surfaces are plotted in Fig. 2b with *s* strongly affecting the experimental outcome (see Supplementary Fig. 2 for more details). Suffusion occurred exclusively for the texture with the smallest pitch, with impalement conversely only observed for the sparser pillar spacings, and expulsion occupying the intermediate region. In contrast to the importance of *s*, varying *h* had (particularly for small pitch values) little impact on *Φ* (Fig. 2b). Interestingly, unique to the impalement case, we noticed a dependency of *Φ* on *β* (Fig. 2c). Here, impalement was almost exclusively observed for *β* < 90° (for an explanation of impalement for *β* ≥ 90°, see Supplementary Fig. 3). This suggests that the nucleation-induced symmetry breaking of the droplet during recalescence contributes to this mechanism. Previous work has determined that the evaporation rate of a droplet increases substantially during recalescence^22,23^. We therefore propose, and subsequently substantiate with local pressure measurements and a theoretical analysis, that the asymmetric recalescent freezing of the droplet leads to an asymmetric explosive release of vapour, engendering a reaction force directed away from the point of nucleation. We term this the recalescence force with a magnitude of *F*_r_, which arises as a difference in the vapour flux from the recalesced (*j*_r_) and supercooled liquid (*j*_c_) phases, whose maximum value is expressed as:

1 $$F_{{{\mathrm{r}}}} = \uppi R_{\mathrm{d}}^2\left( {j_{{{\mathrm{r}}}}^2 - j_{{{\mathrm{c}}}}^2} \right)/\rho _{{{\mathrm{v}}}}$$

where *R*_d_ is the droplet radius and *ρ*_v_ is the vapour density (see ‘Theoretical vapourisation force’ in the Supplementary Information)^35^.

Using dedicated pressure measurements and modelling of droplet evaporation including kinetic and diffusive resistances to vapour transport^36^, we quantified the vapour fluxes (*j*_r_ and *j*_c_), as well as the pressure increase in the vicinity of the freezing droplet, Δ*p*_r_, using miniaturized pressure sensors (see ‘Pressure measurement’ in the Supplementary Information and Supplementary Figs. 4 and 5). Over a total of 46 experiments, we sampled the transient pressure changes during recalescence and measured Δ*p*_r_ to be on average 14 Pa with a standard deviation of 4 Pa. By assuming that $\Delta p_{\mathrm{r}} = \left( {j_{\mathrm{r}}^2-j_{\mathrm{c}}^2} \right)\rho _{\mathrm{v}}^{{{{\mathrm{ - 1}}}}}$, equation (1) can be re-expressed as $F_{\mathrm{r}}{{{\mathrm{ = }}}}\uppi R_{\mathrm{d}}^2\Delta p_{\mathrm{r}}$. Both approaches (based on the vapour fluxes and based on Δ*p*_r_) lead to consistent results for *F*_r_ of approximately 100 μN for a 10 μl droplet (see ‘Vapour flux’ in the Supplementary Information, Supplementary Fig. 6 and Supplementary Table 2). In our further force analysis, we neglected any overpressure resulting from vapour drainage through the texture, as reported for steady evaporation in previous work^20^. This is justified due to the unsteady sudden increase of the evaporation during recalescence, which dwarfs any steady evaporation, and is further substantiated by our experimental observations of no notable influence of *h* on *Φ*, as well as robust expulsion events on porous textures where no overpressure can be built (Supplementary Fig. 7 and Supplementary Video 4).

To understand how the enhanced vapour flux surrounding recalescence influenced the outcomes observed, we theoretically considered the other dominant forces exerted on the freezing droplet. Controlling the interaction between droplet and substrate are an adhesion force, *F*_a_, and a capillary force, *F*_c_, both acting normal to the substrate. *F*_a_ is derived from the practical work of adhesion for the case of a droplet resisting vertical removal from a substrate and is defined as *F*_a_ = 2π*R*_c_*σ*(1 + cos*θ*_r_) (see ‘Adhesion force’ in the Supplementary Information and Supplementary Fig. 8)^16,37,38^. Here, *σ* is the surface tension of water at 0 °C (see ‘Surface tension’ in the Supplementary Information), *θ*_r_ is the receding contact angle and *R*_c_ = *R*_d_sin*θ*_*e*ff_ is the droplet contact radius with the substrate, which is a function of the effective droplet contact angle *θ*_*e*ff_ computed as the average of the advancing and receding contact angles (Supplementary Table 1). The capillary force resisting impalement of the droplet into the texture is defined as $F_{{{\mathrm{c}}}} = \uppi R_{{{\mathrm{c}}}}^2p_{{{\mathrm{c}}}}\left( {1 - f} \right)$, where *p*_c_ is the capillary pressure and *f* is the wetting fraction. For cylindrical micropillars, $p_{{{\mathrm{c}}}} = - \left( {2f/\left( {1 - f} \right)} \right)\cos \theta _{{{\mathrm{c}}}}\left( {2\sigma /d} \right)$ and $f = {\uppi}d^2/4s^2$, where *θ*_c_ is the advancing contact angle on the sides of the pillars^27^ (see ‘Capillary pressure’ and ‘Surface property effects’ in the Supplementary Information and Supplementary Fig. 9). Both *F*_a_ and *F*_c_ were modelled as liquid–solid interactions because we experimentally observed mostly liquid-like behaviour at the droplet–substrate interface during recalescence—even in cases for which nucleation initiates from said interface (see ‘Adhesion force’ in the Supplementary Information and Supplementary Fig. 10). Nucleation from the droplet–substrate interface would be more representative of freezing in a high-humidity environment in which nucleation from the free surface is less favourable (see ‘Relative humidity’ in the Supplementary Information).

**Fig. 3:**
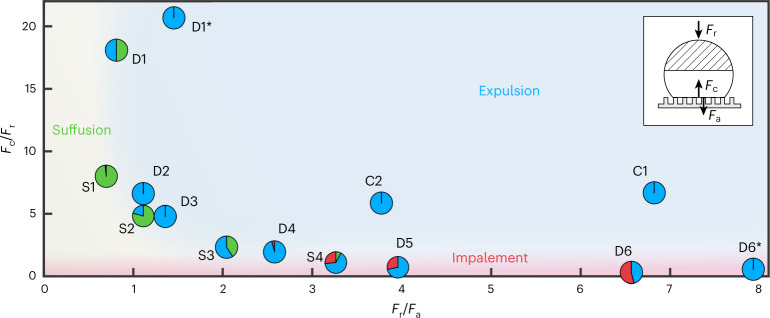
Force scaling analysis of freezing-induced wetting transition mechanisms. *F*_c_/*F*_r_ versus *F*_r_/*F*_a_ for the micropillar surfaces with constant diameter (D1 to D6), the micropillar surfaces with constant pitch (S1 to S4) and the spray-coated glass (C1) and mesh (C2), as well as the spray-coated micropillar surfaces (D1* and D6*) evaluated for the low-pressure conditions. The pie charts indicate the probabilities of the three different outcomes (red, impalement; blue, expulsion; green, suffusion), while the centre point of the pie charts locates the respective samples in the force ratio map evaluated for an initial water droplet volume of *V* = 10 μl. The background shading serves as a guide to the eye to identify regions of the three outcomes. *N* = 651, *n* ≥ 10. *V* = 10 μl for 461 experiments, while *V* ∈ [2 20] μl for the remaining 190 experiments. The effect of *V* is minor (see ‘Droplet size’ in the Supplementary Information and Supplementary Fig. 16). See Supplementary Tables 1 and 3 for further details. The inset is a schematic of a droplet on a micropillar surface with the dominant forces labelled. Source data

In Fig. 3, we show the computed ratios of the introduced forces for 14 superhydrophobic surfaces against the outcomes of *N* = 651 freezing events (Supplementary Tables 1 and 3 and Supplementary Fig. 11). From this, we verified that the freezing outcome is indeed determined by a competition between the two resisting forces (*F*_a_ and *F*_c_) and *F*_r_. For droplets of this size, gravity plays a minor role in the outcome of a freezing event and is therefore neglected for the following analysis (see ‘Gravity’ in the Supplementary Information). Comparing these forces with one another, we can see that both conditions of (*F*_r_/*F*_a_) > 1 and (*F*_c_/*F*_r_) > 1 must be satisfied for expulsion to reliably occur. However, if (*F*_r_/*F*_a_) < 1, post-recalescence crystallization takes place while the droplet is still in contact with the surface and the suffusion of slush into the texture from within the contact area is observed (Fig. 1e,f). Suffusion can be explained by excretion of slush from the core to relieve internal stresses built up by volumetric expansion during inward solidification in a similar manner to the mechanism responsible for self-dislodging^24^. Analogously, (*F*_c_/*F*_r_) < 1 can lead to impalement of the droplet onto the texture; that is, a permanent transition from Cassie–Baxter to Wenzel wetting. Therefore, to promote expulsion as a means of achieving icephobicity, a substrate must simultaneously minimize *F*_a_ and maximize *F*_c_. However, for single-tier surface textures including the micropillars used here, a monotonic relationship exists between these two forces leading to the asymptotic trend in the force ratios when plotted for varying *s* or *d*. Consequently, to produce robust icephobic surfaces, it is necessary to decouple *F*_a_ from *F*_c_. This can be achieved by introducing additional layers of texturing, as we demonstrate with the preparation of spray-coated micropillar (D1*, D6*), glass (C1) and mesh (C2) substrates, which contain layers of nano- and microtexture (as well as a macrotexture in the case of the mesh). The asterisks in the micropillar designations denote modification of the underlying PDMS samples used previously, which we realized by spray coating a conformal layer of hydrophobic nanoparticles (Methods). Applying the spray coating to sample D1 substantially increased *θ*_r_ through a reduction in *f*, and thereby also enhanced (*F*_r_/*F*_a_) from 0.8 (for D1) to 1.5 (for D1*). The change in the force ratio was reflected in the experimental outcome: while D1 showed an expulsion probability of 50% and otherwise suffusion, D1* always provided expulsion. Applying the spray coating to sample D6 doubled (*F*_c_/*F*_r_) from 0.3 (for D6) to 0.6 (for D6*) due to the increased *θ*_c_. Although D6 suffered from an impalement probability of more than 50%, D6* exhibited expulsion in all cases, irrespective of *β*, despite a potential temporary transition into a micro-Wenzel state during recalescence (Supplementary Fig. 12 and Supplementary Video 5). We suggest that the conformal nanoparticle coating allows the droplet to maintain a Cassie state at the nanometre scale and recover from the micrometre-scale wetting transition^39^. We note that, when defining the force ratios, we were simplifying the analysis by focusing on the magnitude of *F*_r_ and not accounting for the effect of *β* on the relevant normal component of *F*_r_, which could be compensated for with a multiplication factor of cos(*β*) (Fig. 2a). We justify this omission by considering that *Φ* is independent of *β* for suffusion (Fig. 2c) and that the absolute magnitude of *F*_r_ represents a rational and conservative estimate in the case of impalement (cos(*β*) ≤ 1), since functional icephobic surfaces should resist impalement for all possible *β*.

Furthermore, we have demonstrated the validity of the proposed modelling and the robustness of the observed phenomena by performing freezing experiments on geometrically equivalent silicon micropillar surfaces that have a thermal conductivity and elastic modulus more than four orders of magnitude higher than PDMS (see ‘Substrate thermal conductivity and elastic modulus’ in the Supplementary Information and Supplementary Fig. 13), as well as with droplets composed of aqueous solutions of glycerol and a surfactant, respectively (see ‘Surfactant effect’ in the Supplementary Information and Supplementary Figs. 14 and 15). We also found that the observed phenomena were barely affected by variation in *V*, which agreed with our modelling that predicted (*F*_r_/*F*_a_) ∼ *V*^1/3^ and (*F*_c_/*F*_r_) to be independent of *V* (see ‘Droplet size’ in the Supplementary Information and Supplementary Fig. 16).

**Fig. 4:**
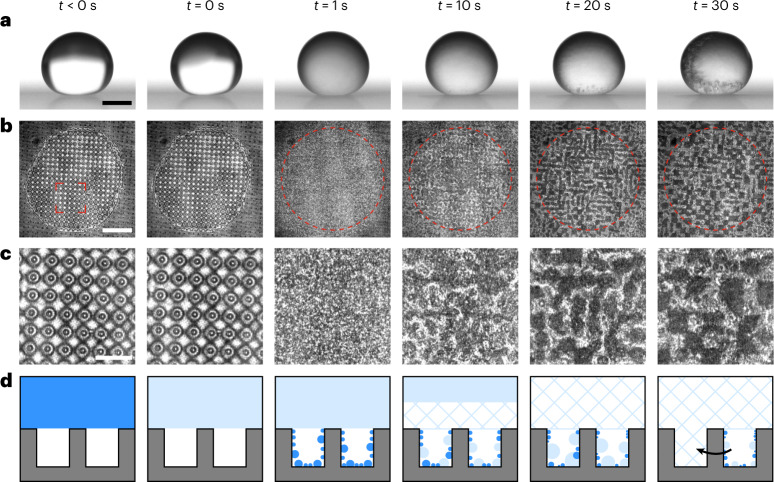
Freezing on superhydrophobic surfaces at ambient pressure. **a**,**b**, Synchronized side- (**a**) and bottom- (**b**) view image sequences of a water droplet freezing in a cold, dry environment at atmospheric pressure (red circles mark the approximate location of the contact line post-recalescence) on a superhydrophobic PDMS texture [*d*, *s*, *h*] = [10, 50, 40] µm (identifier D2 in Supplementary Table 1). **c**, Enlarged view between the pillars underneath the droplet for each timestep (for the regions of interest marked in **b**). **d**, Schematic of the bottom-up suffusion mechanism responsible for surface failure from condensation filling, coalescence (black arrow) and freezing. Water, ice slush and solid ice are represented by blue shading, light blue shading and hatching, respectively. Scale bars: **a**, 1 mm; **b**, 300 µm; **c**, 100 µm. All experiments (*N* = 24) showed bottom-up suffusion. Employed surfaces: D2 and D5 with pillar heights of 25 and 40 μm (*n* = 6) (see Supplementary Table 1 for details).

To complete our understanding of recalescence on superhydrophobic surfaces, we explored the reciprocal case of freezing at ambient pressure (chamber pressure *P* ≈ 96 kPa, RH ≈ 0%; see ‘Relative humidity’ in the Supplementary Information for the effect of RH) and low temperature (surface temperature, *T*_s_ ≈ *T*_∞_ ≈ −20 °C; isothermal conditions). Figure 4a–c presents side- and bottom-view images of a droplet freezing on a micropillar substrate that reliably yielded expulsion under low-pressure conditions (Supplementary Video 6). However, we observed no movement of the droplet during recalescence, instead seeing condensation form within the texture approximately 1 s after ice nucleation. These condensate nuclei grow and coalesce within the texture before freezing (indicated by the disappearance of interference fringes). When nuclei from neighbouring cells coalesce, they are pinned by the microtexture and do not move laterally^40^, instead growing upwards and emerging from the texture to connect with the frozen droplet. This is in contrast to observations of droplets interacting with multitier nanotextured substrates, where spontaneous motion was observed upon coalescence^7,10^. Fresh vapour can then condense in the space left behind after coalescence. This mechanism of bottom-up suffusion of the texture is further illustrated in Fig. 4d. The degree of coalescence observed is dependent on the dimensions of the texture, but there is sufficient vapour released during a freezing event for ice to bridge from the bottom of the texture to the frozen droplet irrespective of the pillar pitch (Supplementary Fig. 17). We observed bottom-up suffusion irrespective of nucleation location with nucleation at the droplet–substrate interface observed for 8 of the *N* = 24 experiments performed; we note that this would be the expected nucleation location for freezing events in a high-humidity environment (see ‘Relative humidity’ in the Supplementary Information).

To explain the presence of a distinct mechanism at ambient pressure, it is necessary to consider the implications of the environmental conditions on the freezing process. We have already ascribed the motion of the droplet to a bias in the explosive release of vapour during recalescence, and have experimentally and theoretically determined its magnitude in the low-pressure environment. For the ambient-pressure experiments, the Fickian diffusion resistance dominates evaporation, such that *j* ∼ *D*_v_, where *D*_v_ is the vapour diffusivity that depends on the chamber pressure as *D*_v_ ∼ *P*^−1^ (see ‘Vapour flux’ in the Supplementary Information)^41^. As *P* in the low-temperature, atmospheric-pressure case is approximately three orders of magnitude larger than in the low-pressure conditions, *j*_r_ and *j*_c_ are accordingly three orders of magnitude smaller and the corresponding *F*_r_ is reduced by six orders of magnitude (equation (1)).

Instead of propelling the droplet, the vapour released now permeates the texture and condenses on the substrate, because after recalescence the droplet temperature (*T*_d_) exceeds *T*_s_. This behaviour is in complete contrast to the low-pressure experiments where there is the same rise in *T*_d_ over recalescence but is still lower than that of the substrate and ambient environment. This temperature difference has also been observed to give rise to condensation and subsequently the frost halo phenomenon^22^, as well as the partial penetration of metastable ice menisci within superhydrophobic surfaces (via capillary condensation) responsible for increased ice adhesion^42,43^. Other works have observed reductions in contact angles^44^, condensation^45^ and freezing^46^ within the texture beneath cooled sessile droplets but, crucially, have relied on the substrate being sub-cooled relative to the droplet or the environment. Another consequence of the diminished *j* at atmospheric pressure is an increase in the time period required for the droplet to thermally re-equilibrate with the surroundings due to a reduction in the rate at which heat from the droplet is rejected through evaporation to the environment that is not compensated for by heat conduction into the cold substrate. This slows the crystallization phase of freezing and explains the persistence of condensate formation long after recalescence has ceased (see ‘Thermal profile of a freezing droplet’ in the Supplementary Information and Supplementary Fig. 18). To prevent condensate filling the intervening air layer, which is the initial step of bottom-up suffusion, it would therefore be necessary to alter the surface texture in such a way as to prevent nucleation^46^ or, alternatively, promote motion of the formed condensate droplets out from between the asperities using a Laplace pressure imbalance before freezing can occur^7,47^.

In this work, we have exposed and explained three outcomes of the freezing of water droplets deposited on superhydrophobic surfaces under low-pressure conditions. Through design of the substrate texture, measurement of the vapour released during recalescence and formulation of a theoretical model, we have established that a bias in the explosive evaporative flux of a freezing droplet results in a force that can expel the droplet from the surface or result in penetration of the droplet into the texture via either droplet impalement or ice suffusion. By considering the driving and resisting forces acting on the droplet during recalescence, we have identified a texture design criterion of simultaneous low surface adhesion and high capillary pressure as being essential to achieving robust droplet expulsion, and demonstrated this by performing experiments on rationally engineered hierarchical surfaces. Furthermore, we have explored the complementary freezing regime of low temperature and atmospheric pressure, revealing a mechanism of surface anti-icing failure through condensation, freezing and bottom-up suffusion of the surface texture that is distinct from those seen under low pressure. This behaviour, a direct consequence of phase change and therefore endemic to all freezing events requiring some degree of supercooling, was explained through consideration of the effect of ambient pressure on diffusion and evaporation. The surprising results of the phenomena documented here could have far-reaching implications for the application of superhydrophobic technologies to the design of icephobic surfaces across a large spectrum of environmental pressure and temperature conditions spanning sectors including transportation, energy and infrastructure.

## Methods

### Materials

PDMS silicone elastomer and curing agent (Sylgard, 10:1 ratio) were purchased from The Dow Chemical Company. For the spray-coating preparation, we used 1-methyl-2-pyrrolidinone (NMP, Sigma-Aldrich), poly(methyl methacrylate) (PMMA) powder (Sigma-Aldrich), poly(vinylidene fluoride) (PVDF) pellets (Sigma-Aldrich), acetone (Thommen-Furler AG) and hydrophobic fumed silica nanoparticles (HFS, Evonik). As substrates we used glass microscope slides (1 mm thickness, VWR) and woven stainless steel meshes (wire diameter 90 µm, mesh opening 190 µm; TWP Inc.).

### Preparation

To fabricate transparent micropillars, we produced a silicon negative of the desired texture using the deep reactive-ion etching Bosch process. PDMS and the curing agent were mixed by hand at a ratio of 10:1 and subsequently degassed in a vacuum chamber. The resulting mixture was then poured over the silicon negative and degassed again before being cured in an oven at 70 °C for 2 h. When fully cured, the PDMS positive was peeled from the silicon and samples were cut to size using a scalpel.

We employed two different spray coatings. One coating contained PVDF, PMMA and HFS, and we refer to it as Coating A. The other coating contained only HFS and we refer to it as Coating B. To prepare the mixture for Coating A, we used the same recipe as in ref. ^48^. First, we prepared separate stock solutions of 10 wt% PVDF in NMP and 10 wt% PMMA in acetone by dissolving the polymers under mechanical mixing for 12 h at 50 °C on a hot plate and at room temperature, respectively. Using probe sonication three times for 30 s, we suspended 1.16 g HFS in 16.88 g acetone. Subsequently, we added 1 g PMMA-in-acetone stock solution and 1 g PVDF-in-NMP stock solution. Finally, we mixed all of the components by shaking. This spray-coating mixture was used to coat glass microscope slides and stainless steel meshes. First, we cut the meshes to the desired sample size of a few square centimetres. We then cleaned the microscope slides and the meshes with isopropyl alcohol and dried them with pressurized nitrogen gas. In a next step, the samples were spray coated with the nanoparticle coating using a siphon-feed airbrush (Paasche; back pressure ~2 bar, spray distance ~15 cm). Meshes were coated from both sides. After completing the spray coating, the samples were dried for 10 min on a hot plate at 100 °C to remove residual solvents. To modify the PDMS samples with Coating B, we suspended 0.1 g HFS in 8 g acetone and probe sonicated as described above. Spray coating was then performed similarly with a back pressure of ~3 bar. The samples were then dried as above.

### Characterization

The surface structures were characterized with both optical (Olympus BX60) and scanning electron microscopy (Hitachi SU8230). All contact angle measurements were performed with a DataPhysics OCA 35 goniometer (three independent measurements each).

### Experimental set-up and protocols

In the case of the low-pressure experiments, a sessile droplet was deposited onto a superhydrophobic sample in the Cassie–Baxter state using an Eppendorf pipette. The experimental chamber (Supplementary Fig. 19) was then closed and purged of water vapour by flushing dry gaseous nitrogen through until the humidity sensor (LinPicco Basic A05-G) read 0% RH. The environmental chamber was then evacuated to reach a chamber pressure *P* ≈ 0.1 kPa until the droplet spontaneously froze (typically after around 15 s). The chamber temperature as measured by a sensor at a distance of about 5 cm from the droplet remained practically constant across a freezing event at 22 ± 1 °C. Convection related to chamber pump-down did not affect the observed phenomena (see Supplementary Fig. 20 and Supplementary Video 7). After repressurization, the frozen droplet was removed, and the sample dried in situ with a burst of dry gaseous nitrogen.

For ambient-pressure experiments, a continuous flow of evaporated liquid nitrogen from a Kaltgas system was passed through the environmental chamber and base producing a dry (RH ≈ 0%), isothermal environment simultaneously cooling the gas phase and the substrate to the desired temperature (see ‘Relative humidity’ in the Supplementary Information for the effect of RH and Supplementary Fig. 19). A deflector was used to prevent the flow from shearing the droplet and affecting the freezing behaviour. Droplets were allowed to thermalize on the tip of a syringe inside the chamber before being deposited on the surface in the Cassie–Baxter state. Following freezing (typically after around 60 s), the frozen droplet was removed and the surface dried as before. The chamber was then allowed to reach thermal equilibrium again before another experiment was performed.

Freezing events were visualized from both the side and bottom (through an inverted interference reflection microscope) synchronously using Photron UX-mini and SA1.1 high-speed cameras, respectively, at a rate of 5,000 frames per second^46,49^. The side view was illuminated by a diffuse white light-emitting diode source (Advanced Illuminations) and the bottom view by a pigtail laser diode (peak wavelength 633 nm; Thorlabs). All sensor data were obtained and synchronized with the cameras using a data acquisition board (National Instruments).

## Online content

Any methods, additional references, Nature Portfolio reporting summaries, source data, extended data, supplementary information, acknowledgements, peer review information; details of author contributions and competing interests; and statements of data and code availability are available at 10.1038/s41567-023-01946-3.

## Supplementary information

Supplementary Information Supplementary Figs. 1–20, Tables 1–3 and Discussion.

Supplementary Video 1 Impalement of a supercooled droplet during freezing on a superhydrophobic substrate in a low-pressure environment.

Supplementary Video 2 Rapid expulsion of a supercooled droplet during freezing on a superhydrophobic surface in a low-pressure environment.

Supplementary Video 3 Top-down suffusion of a post-recalescence droplet into a superhydrophobic surface in a low-pressure environment.

Supplementary Video 4 Expulsion event on a porous superhydrophobic mesh in a low-pressure environment.

Supplementary Video 5 Expulsion event on an HFS-modified hierarchical superhydrophobic surface in a low-pressure environment.

Supplementary Video 6 Bottom-up suffusion of ice slush within a superhydrophobic texture beneath a post-recalescence droplet cooled in an atmospheric-pressure, low-temperature environment.

Supplementary Video 7 Expulsion event while the valve between the environmental chamber and vacuum pump is closed, thereby excluding a potential effect of convection on droplet nucleation.

## Data Availability

All data used to produce this Letter can be found in the ETH Research Collection at 10.3929/ethz-b-000539612. Original videos files too large to be uploaded are available from the corresponding authors upon reasonable request. Source data are provided with this paper.

## Source data

Source Data Figs. 2 and 3 Outcomes and experimental variable for all 249 experiments plotted in Fig. 2b,c and experimental overview for data plotted in Fig. 3.

## Code availability

All codes used to produce this Letter can be found in the ETH Research Collection at 10.3929/ethz-b-000539612.

## References

[1] Schutzius TM (2015). Physics of icing and rational design of surfaces with extraordinary icephobicity. Langmuir.

[2] Nath S, Ahmadi SF, Boreyko JB (2017). A review of condensation frosting. Nanosc. Microsc. Thermophys. Eng..

[3] Kreder MJ, Alvarenga J, Kim P, Aizenberg J (2016). Design of anti-icing surfaces: smooth, textured or slippery?. Nat. Rev. Mater..

[4] Tourkine P, Le Merrer M, Quéré D (2009). Delayed freezing on water repellent materials. Langmuir.

[5] Mishchenko L (2010). Design of ice-free nanostructured surfaces based on repulsion of impacting water droplets. ACS Nano.

[6] Maitra T (2014). On the nanoengineering of superhydrophobic and impalement resistant surface textures below the freezing temperature. Nano Lett..

[7] Boreyko JB, Collier CP (2013). Delayed frost growth on jumping-drop superhydrophobic surfaces. ACS Nano.

[8] Irajizad P, Hasnain M, Farokhnia N, Sajadi SM, Ghasemi H (2016). Magnetic slippery extreme icephobic surfaces. Nat. Commun..

[9] Eberle P, Tiwari MK, Maitra T, Poulikakos D (2014). Rational nanostructuring of surfaces for extraordinary icephobicity. Nanoscale.

[10] Boinovich LB, Emelyanenko AM, Emelyanenko KA, Modin EB (2019). Modus operandi of protective and anti-icing mechanisms underlying the design of longstanding outdoor icephobic coatings. ACS Nano.

[11] Boinovich L, Emelyanenko AM, Korolev VV, Pashinin AS (2014). Effect of wettability on sessile drop freezing: when superhydrophobicity stimulates an extreme freezing delay. Langmuir.

[12] Wong T-S (2011). Bioinspired self-repairing slippery surfaces with pressure-stable omniphobicity. Nature.

[13] Golovin K (2016). Designing durable icephobic surfaces. Sci. Adv..

[14] Irajizad P (2019). Stress-localized durable icephobic surfaces. Mater. Horiz..

[15] Golovin K, Dhyani A, Thouless MD, Tuteja A (2019). Low–interfacial toughness materials for effective large-scale deicing. Science.

[16] Meuler AJ (2010). Relationships between water wettability and ice adhesion. ACS Appl. Mater. Interf..

[17] Chen J (2013). Robust prototypical anti-icing coatings with a self-lubricating liquid water layer between ice and substrate. ACS Appl. Mater. Interf..

[18] Subramanyam SB, Rykaczewski K, Varanasi KK (2013). Ice adhesion on lubricant-impregnated textured surfaces. Langmuir.

[19] Jung S (2011). Are superhydrophobic surfaces best for icephobicity?. Langmuir.

[20] Jung S, Tiwari MK, Doan NV, Poulikakos D (2012). Mechanism of supercooled droplet freezing on surfaces. Nat. Commun..

[21] Schutzius TM (2015). Spontaneous droplet trampolining on rigid superhydrophobic surfaces. Nature.

[22] Jung S, Tiwari MK, Poulikakos D (2012). Frost halos from supercooled water droplets. Proc. Natl Acad. Sci. USA.

[23] Graeber G, Dolder V, Schutzius TM, Poulikakos D (2018). Cascade freezing of supercooled water droplet collectives. ACS Nano.

[24] Graeber G, Schutzius TM, Eghlidi H, Poulikakos D (2017). Spontaneous self-dislodging of freezing water droplets and the role of wettability. Proc. Natl Acad. Sci. USA.

[25] Wildeman S, Sterl S, Sun C, Lohse D (2017). Fast dynamics of water droplets freezing from the outside in. Phys. Rev. Lett..

[26] Cassie ABD, Baxter S (1944). Wettability of porous surfaces. Trans. Faraday Soc..

[27] Bartolo D (2006). Bouncing or sticky droplets: impalement transitions on superhydrophobic micropatterned surfaces. Europhys. Lett..

[28] Papadopoulos P, Mammen L, Deng X, Vollmer D, Butt H (2013). How superhydrophobicity breaks down. Proc. Natl Acad. Sci. USA.

[29] Lafuma A, Quéré D (2003). Superhydrophobic states. Nat. Mater..

[30] Antonini C (2015). Unraveling wetting transition through surface textures with X-rays: liquid meniscus penetration phenomena. Sci. Rep..

[31] Wenzel RN (1936). Resistance of solid surfaces to wetting by water. Ind. Eng. Chem..

[32] Moulinet S, Bartolo D (2007). Life and death of a fakir droplet: impalement transitions on superhydrophobic surfaces. Eur. Phys. J. E.

[33] Burton JC, Sharpe AL, van der Veen RCA, Franco A, Nagel SR (2012). Geometry of the vapor layer under a Leidenfrost drop. Phys. Rev. Lett..

[34] Panchanathan D (2021). Levitation of fizzy drops. Sci. Adv..

[35] Cazabat A-M, Guéna G (2010). Evaporation of macroscopic sessile droplets. Soft Matter.

[36] Vaartstra G, Lu Z, Lienhard JH, Wang EN (2022). Revisiting the Schrage equation for kinetically limited evaporation and condensation. J. Heat Transfer.

[37] Gao L, McCarthy TJ (2008). Teflon is hydrophilic. Comments on definitions of hydrophobic, shear versus tensile hydrophobicity, and wettability characterization. Langmuir.

[38] Butt H-J (2017). Energy dissipation of moving drops on superhydrophobic and superoleophobic surfaces. Langmuir.

[39] Verho T (2012). Reversible switching between superhydrophobic states on a hierarchically structured surface. Proc. Natl Acad. Sci. USA.

[40] Dorrer C, Rühe J (2007). Condensation and wetting transitions on microstructured ultrahydrophobic surfaces. Langmuir.

[41] Pruppacher, H. R. & Klett, J. D. *Microphysics of Clouds and Precipitation* (Springer, 2010).

[42] Boinovich L, Emelyanenko AM (2014). Role of water vapor desublimation in the adhesion of an iced droplet to a superhydrophobic surface. Langmuir.

[43] Boinovich LB, Emelyanenko KA, Emelyanenko AM (2022). Superhydrophobic versus SLIPS: temperature dependence and the stability of ice adhesion strength. J. Colloid Interf. Sci..

[44] Chu F, Gao S, Zhang X, Wu X, Wen D (2019). Droplet re-icing characteristics on a superhydrophobic surface. Appl. Phys. Lett..

[45] Oberli L (2014). Condensation and freezing of droplets on superhydrophobic surfaces. Adv. Colloid Interf. Sci..

[46] Lambley H, Schutzius TM, Poulikakos D (2020). Superhydrophobic surfaces for extreme environmental conditions. Proc. Natl Acad. Sci. USA.

[47] Sharma CS, Stamatopoulos C, Suter R, von Rohr PR, Poulikakos D (2018). Rationally 3D-textured copper surfaces for laplace pressure imbalance-induced enhancement in dropwise condensation. ACS Appl. Mater. Interf..

[48] Graeber G, Martin Kieliger OB, Schutzius TM, Poulikakos D (2018). 3D-printed surface architecture enhancing superhydrophobicity and viscous droplet repellency. ACS Appl. Mater. Interf..

[49] Graeber G (2021). Leidenfrost droplet trampolining. Nat. Commun..

