# Supplementary material for: Freezing-induced wetting transitions on superhydrophobic surfaces
Source: Nat Phys. 2023 Feb 9;19(5):649–55. doi: 10.1038/s41567-023-01946-3 (PMC10185467; doi:10.1038/s41567-023-01946-3)
Supplement: Supplementary file 1 — Supplementary Figs. 1–20, Tables 1–3 and Discussion. [file 41567_2023_1946_MOESM1_ESM.pdf]

nature physics

Letter

https://doi.org/10.1038/s41567-023-01946-3

# Freezing-induced wetting transitions on superhydrophobic surfaces

In the format provided by the authors and unedited

Table of Contents

**This PDF file includes:**

Supplementary Information Text
Figures S1 to S20
Tables S1 to S3
Legends for Videos S1 to S7

**Other supplementary materials for this manuscript include the following:**

Videos S1 to S7

## Supplementary Information Text

### Relative Humidity

The experiments presented in this work take place in dry ambient environments because of the experimental setups used. We anticipate that variations in the ambient relative humidity (*RH*) might have implications for the nucleation location on the droplet – which could further influence the capillary and adhesion forces – as well as the evaporation of the recalescing droplet. Here, we present discussions of these effects for both the low- and atmospheric-pressure experiments. Overall, we anticipate that all our phenomena are largely insensitive to the environmental *RH*.

Nucleation Location

An increase in the ambient relative humidity would reduce the vaporisation rate from the droplet free surface, which would favour nucleation from the droplet-substrate interface. This could result in a higher adhesion force post-recalescence, e.g., due to ice anchoring to the surface, than the complementary case where nucleation occurs from the droplet-air interface.

In our atmospheric-pressure experiments, we observed nucleation from both the free surface (N = 8, 33% of experiments) and the droplet-substrate interface (N = 16, 66% of experiments) in a dry ($RH \approx 0$) environment. The bottom-up suffusion mechanism occurred in all cases, regardless of nucleation location, suggesting that the phenomenon would be insensitive to variations in *RH*.

In our low-pressure experiments, the droplet cools exclusively due to evaporation at the free surface, making nucleation from the droplet-air interface favourable. Even if the atmosphere was pure water vapour, the difference in vapour pressure between that above the surface of the supercooled droplet (which we calculate to be 191.3 Pa at -15°C, see Table S2) and the environment (on average 72 Pa in the experiments presented in Fig. S4) would be sufficient such that strong evaporation would still occur, and homogenous nucleation (at the free surface due to evaporative cooling) would still be the dominant mode of nucleation. Notably, when heterogenous nucleation occurred in 4 out of the N = 249 experiments performed for Fig. 2, we always observed expulsion, which suggests that the nucleation position has little effect on the adhesion of the droplet to the substrate (see SI Adhesion Force and Fig. S10 for more details).

Evaporation

In both experimental conditions, the presence of ambient water vapour will reduce the evaporation rate form the droplet.

In our atmospheric-pressure experiments, the central factor determining condensation is the local supersaturation under the recalesced droplet. This local supersaturation is computed as the ratio of the vapor pressure at the droplet free surface after recalescence and the saturated vapor pressure above the cold substrate. It is important to note that both vapour pressures do not depend on *RH*. Instead, they are fixed by the droplet temperature after recalescence ($T_d$ = 0 °C) and the temperature of the substrate ($T_s$ = -20 °C). Therefore, it can be assumed that the obtained results

on bottom-up suffusion under atmospheric-pressure conditions are valid for all values of *RH*, ranging from dry (0%) to saturated (100%).

In our low-pressure experiments, the recalescence force $F_r$ that drives droplet motion, results from the rapid, asymmetric release of vapor from the droplet during recalescence. Based on Equation (1) in the main text, $F_r$ is proportional to $(j_r^2 - j_c^2)$, where $j_r$ and $j_c$ are the vapour flux of the recalesced and the supercooled liquid phase of the droplet, respectively. Since $j_r^2 >> j_c^2$, for the purposes of the following analysis this can be simplified to $F_r \sim j_r^2$. Furthermore, for evaporation, $j_r \sim (p_{v,surface} - p_{v,\infty})$, where $p_{v,surface}$ is the vapour pressure at the free surface of the droplet, and $p_{v,\infty}$ is the vapour pressure in the environment. $p_{v,surface}$ has a constant value of 611 Pa, since it is the saturated vapour pressure of water at the equilibrium freezing temperature. In contrast, $p_{v,\infty}$ scales with *RH* and the total pressure in the chamber, $p_{tot}$, such as $p_{v,\infty} = RH \cdot p_{tot}$. In the low-pressure experiments, we measured $p_{tot}$ to be on average 72 Pa at the moment of recalescence freezing. In summary, $F_r \sim (p_{v,surface} - RH \cdot p_{tot})^2$. To understand the effect of RH, we compute a ratio of $F_r$ ($RH$ = 100%) / $F_r$ ($RH$ = 0%) = $(p_{v,surface} - p_{tot})^2 / p_e^2$ = 78%. This shows that $F_r$ only mildly reduces (by 22%) when increasing *RH* from 0% (as it is in our experiments) to the maximum value of 100%. The effect of *RH* is therefore minor for these experiments and is not expected to markedly affect the experimental outcome, since simultaneously $F_a$ and $F_c$ do not depend on *RH*. Furthermore, our analysis shows a clear pathway to ensure droplet expulsion at high *RH*, since it is the competition between $F_r$ and $F_a$ that determines occurrence of expulsion. For more humid conditions where $F_r$ is reduced, also $F_a$ needs to be reduced, which is feasible based on appropriate surface engineering (as also shown in the main text based on hierarchical structured surfaces).

## Theoretical Vapourisation Force

Droplet motion is observed to initially occur in a direction opposite to that of the nucleation site. This can be attributed to the enhanced vapourisation of the droplet during recalescence.[1,2] We can estimate the recoil force acting on the droplet, $F_r$, by applying conservation of linear momentum to the ejected vapour mass. The equation of motion for a variable mass system such as this can be expressed by

$$F = \dot{m}v + m\dot{v} - \dot{m}u = -F_r \tag{1}$$

where $u$ and $v$ denote the velocities of the vapour and droplet respectively. As the droplet is at rest initially, Equation (1) simplifies to

$$F_r = \dot{m}u \,. \tag{2}$$

The vapour mass flux can be calculated as

$$\dot{m} = jA = \rho_v A u \tag{3}$$

where $j$ is the vapourisation flux per unit area, $A$ is the frozen surface area and $\rho_v$ is the vapour density. Substituting this into Equation (2) and dividing through by $A$ yields the pressure exerted by the vapour released during recalescence

$$p_r = \frac{j^2}{\rho_v} \,. \tag{4}$$

As we are only interested in the components of the recoil force parallel to the nucleation direction, we can express the recoil force as

$$F_r = \int p_r \cos\varphi dA \tag{5}$$

where $\varphi$ is the polar angle from the point of nucleation. Integrating this over the surface of the droplet (approximated as a sphere) as the frozen area increases with time yields

$$F_r = \frac{\pi j^2 R^2}{\rho_v} \sin^2 \varphi(t) \tag{6}$$

which is maximum for $\varphi(t) = 90°$ which physically means that half the droplet has recalesced. This is expected as for vapour emitted beyond $\varphi(t) > 90°$, the direction of the velocity component reverses.

## Pressure Measurement

Owing to difficulties in estimating $j_r$ due to the inherently unsteady, explosive nature of the evaporation, we decided to collect experimental data to quantify $F_r$. To this end, we measured the local pressure field in the vicinity of the freezing droplet using two miniaturised pressure sensors (Fig. S4a) with measuring cavities similar in size to that of the droplet. We note that the proximity of the closer sensor is bounded by the reduction in size of the droplet due to evaporation prior to nucleation (Fig. S4b). The typical response signals of both sensors (Fig. S4c) are similar with the pressure measured close to and farther from the droplet ($p_1$ and $p_2$, respectively). These signals show pronounced pressure rises during and immediately after recalescence and after reaching plateau values, they slowly decrease at later times. The difference in magnitude between $p_1$ and $p_2$ can be explained by the pressure gradient from the vapour source to the vacuum outlet.

On closer inspection of the region around the time $t = 0$, which is defined as the time at which the $p_1$ sensor responds to the increased vapour flux from the droplet due to recalescence (Fig. S4d), it becomes apparent that, close to the droplet, the greatest rate of pressure increase happens over the timescale of recalescence. Further away from the droplet (~ 20 mm), the $p_2$ pressure response is smoothed by gas diffusion, which also induces a slight delay in the reaction of the second sensor (~ 1 ms). To isolate the steepest part of this rise, which we define as $\Delta p_r$, we iteratively take a linear fit of the pressure response for each timestep from $t = 0$ until the $p_1$ value deviates from a corridor of 99.95% confidence from this fit. In Fig. S4e, we plot $\Delta p_r$ *vs.* $p_{min}$ (the value of $p_1$ at $t = 0$) for each experiment performed. The stochastic nature of nucleation and, therefore, the time between vacuum pull and freezing, leads to a distribution of $p_{min}$ values for the same experimental protocol. Over the 46 experiments, we measure $\Delta p_r$ to be on average 14 Pa with a standard deviation of 4 Pa and no prominent dependence on $p_{min}$.

The measurements of the vapour pressure in the vicinity of the droplet during recalescence were made with a micro-Pirani (Posifa PVC1000) pressure sensors. Pirani sensors measure pressure by using a heated wire element which exchanges thermal energy with the environment. Due to this, they are affected by both the composition and the temperature of the present gas. The sensors were supplied with a constant current with the voltage measured being dependent on the resistance of the thermal element (Ohm's law) whose temperature is dictated by the rate of thermal dissipation to the gas. Due to uncertainties in the gas composition around the droplet during recalescence, calibration curves were made of both dry ($p_{v,\infty}/p_\infty \approx 0$) and wet ($p_{v,\infty}/p_\infty \geq 0.9$) humidity extremes (Fig. S5a) with the final calibration an average of the two (Fig. S5b).

Calibrations with respect to ambient temperature were also performed by cooling the sensors at ambient pressure using evaporated liquid nitrogen (Fig. S5c). Both sensors contain sealed reference resistors which are unaffected by the ambient pressure but have the same temperature response. It was not possible to use these reference resistors during experiments due to the small volume of cold vapour not registering a temperature change. However, the temperature response of each sensor, sealed or not, is intrinsically the same with a constant temperature coefficient of resistance

$$C_{\mathrm{T}} = \frac{1}{R}\frac{dR}{dT} \equiv \frac{1}{V}\frac{dV}{dT} \tag{7}$$

where $R$ is the resistance, $T$ is the temperature and $V$ is the voltage. $C_{\mathrm{T}} = 0.0022\ \mathrm{K}^{-1}$ is calculated from a fit of both sensors and their respective reference resistors (Fig. S5d). The final voltage temperature compensation can therefore be written as

$$\Delta V_{\mathrm{T}} = C_{\mathrm{T}} V_{\mathrm{m}} \Delta T \tag{8}$$

where $V_{\mathrm{m}}$ is the measured voltage and $\Delta T$ is the temperature difference between that of the vapour and the pressure calibration run at room temperature. From previous work, the vapour temperature is close to that of the frozen droplet after recalescence[2] ($\approx 273$ K) and therefore $\Delta T = 20$ K is used to complete the calibration.

## Vapour Flux

In the given environmental conditions where a non-condensable gas (nitrogen) is present, there are two potential resistances to evaporation, namely the kinetic resistance, $\xi_{\text{kinetic}}$, and the diffusive resistance, $\xi_{\text{diffusion}}$.[3] The evaporation rate, $j$, is computed based on these resistances as follows:[3]

$$j = \frac{\Delta p}{\xi_{\text{kinetic}} + \xi_{\text{diffusion}}}$$

where $\Delta p$ is the vapor pressure difference driving the evaporation between the droplet free surface and the continuum vapor region,[4] and is defined as $\Delta p = p_{\text{v},\infty} - p_{\text{v,surface}}$, where $p_{\text{v},\infty}$ is the vapor pressure in the continuum vapor region away from the droplet, and $p_{\text{v,surface}}$ is the vapor pressure at the droplet free surface.

$\xi_{\text{kinetic}}$ characterizes the evaporation from the droplet free surface to the region where the Knudsen layer ends and the continuum vapor region starts.[4] $\xi_{\text{kinetic}}$ occurs within a distance of a few mean free paths from the droplet free surface. An accurate and simple method to compute this resistance is the moment-method.[4] Based on the linearized solution to the moment-method for the vapour flux across the Knudsen layer, we obtain

$$\xi_{\text{kinetic}} = \frac{[\hat{\sigma} + \omega(1 - \hat{\sigma})](2\pi\, R_{\text{H2O}}\, T_{\text{d}})^{0.5}}{\omega\hat{\sigma}}$$

where $\omega = \frac{32\pi}{32+9\pi}$ is a constant, $\hat{\sigma} = 0.62$ is the accommodation coefficient,[5] $R_{\text{H2O}}$ is the specific gas constant of water vapor, and $T_{\text{d}}$ is the temperature of the droplet.

$\xi_{\text{diffusion}}$ characterizes the evaporation between the end of the Knudsen layer into the continuum vapor region and is based on Fickian diffusion. According to Pruppacher and Klett,[6] we can estimate the diffusion coefficient of water vapor in air as

$$D_{\text{V}} = 0.211 \left(\frac{T_{\text{diffusion}}}{273.15\text{ K}}\right)^{1.94} \frac{101325\text{ Pa}}{P}\ \text{cm}^2\text{s}^{-1}\ .$$

where $T_{\text{diffusion}}$ is the average temperature of the gas in which diffusion occurs, and $P$ is the environmental pressure.

Assuming isothermal conditions for simplicity, $\xi_{\text{diffusion}}$ can be expressed based on Fick's diffusion equation as

$$\xi_{\text{diffusion}} = \frac{R_{\text{H2O}}\, T_{\text{diffusion}}\, \Delta x}{D_{\text{V}}}$$

where $\Delta x$ is the diffusion distance (see Table S2).

We distinguish between four main cases for which we compute the evaporation rate, namely, a supercooled droplet in the low-pressure environment, a recalesced droplet in the low-

pressure environment, a supercooled droplet in the ambient-pressure environment and a recalesced droplet in the ambient-pressure environment. We are summarizing the assumptions and results for these four cases in Table S2.

To simplify the computation of the recalescence force, we consider a situation as sketched in Fig. S6. The droplet is a perfect sphere of volume 10 μL. The recalescence freezing front that travels rapidly through the droplet has reached the middle of the droplet, such that the upper hemisphere is recalesced slush (a mixture of water and ice) at the equilibrium freezing temperature, $T_r$ (= 0 °C), whilst the lower hemisphere is still supercooled water at the supercooled temperature, $T_c$, which is approximately -15 °C for the low-pressure experiments and approximately -20 °C for the ambient-pressure experiments. Based on these initial conditions, we can compute the vapour flux for the recalesced upper half of the droplet, $j_r$, and compare it to the flux for the lower half of the droplet that is still a supercooled liquid, $j_c$, as shown in Table S2.

Using these results for $j_r$ and $j_c$ in the low-pressure environment, assuming the vapor to evaporate from the droplet at 0 °C with a density of 0.005 kg m$^{-3}$ into an environment with $RH$ = 0%, we compute $F_r$ = 101 μN, for a 10 μL droplet based on Equation (1) in the main text. This value compares remarkably well to the value of $F_r$, which we obtained through the pressure measurements in Fig. S4, which is $F_r$ = 79 μN. Considering the other possible extreme in $RH$, namely 100%, we obtain $F_r$ = 83 μN. We conclude that $RH$ has a minor effect on $F_r$. For the ambient-pressure environment, we find that diffusion represents a major resistance to the evaporation, thereby also reducing $F_r$ to negligible values on the order of 10$^{-9}$ μN.

**Adhesion force**

We are estimating the adhesion force, $F_a$, between the droplet and the substrate during the first stage of freezing (recalescence) within the first 10 to 20 ms after nucleation. That is supported by our experimental observations, in which expulsion always occurs during the first stage of freezing. Based on an energy balance (assuming adiabatic conditions due to the rapid nature of the event,[1] we can estimate the droplet composition after recalescence as follows.[7] For a droplet with mass, $m_{tot}$, we can compute the mass fraction of ice, $m_{ice}$, after recalescence as, $\mathcal{F} = m_{ice} / m_{tot} = c_w (T_e - T_c) / h_F$ , where $c_w$, $T_e$, $T_c$, and $h_F$ are the heat capacity of liquid water, the equilibrium freezing temperature (0°C), the supercooled droplet temperature (-15°C, see SI, Thermal Profile of a Freezing Droplet, Fig. S18), and the specific enthalpy of fusion, respectively. Substituting appropriate values, we see that $\mathcal{F} \approx 0.2$ at the end of recalescence. The computed value of $\mathcal{F}$ represents an upper bound of the ice mass fraction in the droplet at the moment of expulsion since droplet expulsion is frequently observed before completion of recalescence (see, e.g., Fig. 1). Based on $\mathcal{F}$, we can also estimate the maximum areal fraction of ice present at the droplet/substrate interface as $\Lambda = \{\rho_S\mathcal{F}/[\rho_L(1-\mathcal{F}) + \rho_S\mathcal{F}]\}^{2/3} = 0.33$ in which $\rho_S$ and $\rho_L$ are the densities of ice and liquid water, respectively. Again, $\Lambda$ represents an upper bound of the potential areal contribution of ice to the interactions between the droplet and the substrate. We can conclude that during expulsion the droplet is a slushy mixture of water and ice, but still predominantly liquid (as can be seen in Movie S2).

The nucleation position might play an important role in determining the droplet substrate interactions. To determine if this is the case, we have analysed the ice nucleation location all 249 freezing experiments in Fig. 2. Indeed, we found two distinct cases: droplets that nucleated from the droplet/air interface, which we name homogeneous nucleation, and droplets that nucleated from the droplet/substrate interface, which we name heterogeneous nucleation. Due to the evaporative cooling method that we are applying, homogeneous nucleation is the dominant mode of nucleation with 245 out of 249 analysed experiments.[8]

To better understand our macroscopic experimental observations, we have closely analysed the interfacial dynamics of the freezing droplets. In Fig. S10, we are comparing the two different cases: a droplet showing homogeneous nucleation (Fig. S10a-d) and a droplet showing heterogenous nucleation (Fig. S10e-i). In both cases, we focus on the dynamics of the droplet/substrate interface during the expulsion event. For homogeneous nucleation, the droplet/substrate interface continuously deforms as the droplet de-wets the substrate as highlighted by green arrows in Fig. S10d. The droplet/substrate interface behaves liquid-like throughout the entire dewetting event. These observations can be compared to the heterogeneous nucleation case in Fig. S10e-i. Here, nucleation can be seen to clearly originate from the base of the droplet (as indicated by the blue arrow in **e** and the magnified bottom-view image in **g**). Focussing on the droplet/substrate interface at the moment of expulsion (Fig. S10h,i), we can see that whilst the region of the interface close to the point of nucleation appears rigid (as indicated by the red arrow), further away the interface deforms during and after the expulsion event (green arrow) showing liquid-like behaviour. This suggests that expulsion proceeds more quickly than crystallisation

across the droplet. We therefore conclude that the droplet/substrate interface is behaving mostly in a liquid-like manner even in the case of nucleation from the substrate. Further, we note that the four heterogeneous nucleation cases all resulted in expulsion, thereby not indicating an increase of adhesion force due to heterogeneous nucleation, instead, rather a similar magnitude for both nucleation scenarios (homogeneous and heterogeneous). We can conclude from our experimental observations that the droplet-substrate interface behaves in a liquid-like manner during an expulsion event, even for droplets nucleating from the droplet-substrate interface. It is therefore intuitive and appropriate to model the dewetting process as a liquid being removed from a solid substrate.

Gao and McCarthy[9] establish that the so-called tensile hydrophobicity is best described by the practical work of adhesion that is experienced between a droplet and a solid expressed as $w_p = \sigma(1 + \cos\theta_r)$. This equation is derived by combining the Dupré equation for the work of adhesion between a solid and a liquid ($w_{SL} = \sigma_{SV} + \sigma - \sigma_{SL}$), where $\sigma_{SV}$, $\sigma$, and $\sigma_{SL}$ are the surface tension between solid/vapour, liquid/vapour, and solid/liquid, respectively, and Young's equation relating contact angles and surface tension ($\sigma_{SV} = \sigma_{SL} + \sigma\cos\theta$), where $\theta$ is the respective contact angle. In order to convert $w_p$ into a force of adhesion holding the droplet on the substrate, we consider an infinitesimally small lifting motion of the droplet away from the solid surface as illustrated in Fig. S8. The upward motion leads to a radial dewetting of the droplet along the droplet triple line length $2\pi R_c$ (where $R_c$ is the contact radius) and over a radial distance of $dR_c$ (forming a capillary bridge). The adhesive force resisting this droplet motion is obtained via the basic principle that work done equals the force required to move the contact line multiplied by the distance over which the force is exerted, $w_p\, 2\pi\, R_c\, dR_c = F_a\, dR_c$, which leads to $F_a = 2\pi\, R_c\, \sigma(1 + \cos\theta_r)$. Beyond this simple and general way of assessing $F_a$, there are more sophisticated methods by e.g. Butt et al.[10] that quantify adhesive forces for specific surface geometries and dewetting situations. However, in our present dewetting situation, the employed approach appears sufficiently accurate to understand the trends of the adhesion force as compared to the recalescence force and the capillary force for our scaling analysis. Substituting appropriate values, to obtain an order of magnitude estimation of this force for our experiments, yields $F_a = 20$ μN (considering a 10 μL water droplet on a D5 sample with $R_c = 0.45$ mm, $\sigma = 75.64$ mN m$^{-1}$, $\theta_r = 155°$).

To determine if in our study the presence of ice affects droplet adhesion, we are additionally modelling the case of ice adhesion, which might occur in a portion of the droplet/substrate interface especially as a result from a nucleation location at the droplet/substrate interface. Meuler *et al.* have shown that the practical work of adhesion can be used in a modified way to also estimate ice adhesion.[11] They found that ice adhesion strength, $\tau_{a,S}$, scales as $\sim a\left(1 + \cos\theta_{r,flat}\right)$, where $a = 340 \pm 40$ kPa is an experimentally determined constant of proportionality and $\theta_{r,flat}$ is the intrinsic receding contact angle of the surface without texture. Considering a partially solidified interface between a droplet in a Cassie-Baxter wetting state and a microtextured substrate, which could result from ice nucleation at the droplet/substrate interface at the end of the first stage of freezing, we can compute the contribution of ice to the overall force necessary to remove the droplet from the substrate based on the model of Meuler *et al.* as

$$F_{a,S} \approx \pi\, R_c^2\, \tau_{a,S}\, f\Lambda \approx \pi\, R_c^2 a\left(1 + \cos\theta_{r,flat}\right) f\Lambda,$$

where $f$ is the wetting fraction and $\Lambda$ is the areal solid fraction of ice at the droplet/substrate interface. Inserting appropriate values, we estimate the theoretical maximum contribution of ice adhesion to the overall adhesion force as $F_{a,S}$ = 575 μN ($\theta_{r,flat} = 89°$, $\Lambda = 0.33$, $f = 0.008$; D5 substrate). However, such a high value for $F_a$ would be in disagreement with our experimental observations of droplet expulsion for droplets nucleating from the droplet/substrate interface and to the regime map established in Fig. 3. This can be partially understood by considering that values for $F_a$ obtained with $\Lambda = 0.33$ represent an upper bound of the expected magnitude, since this corresponds to the maximum ice fraction at completion of the first stage of freezing. Furthermore, as observed experimentally in Fig. S10, irrespective of the nucleation position, the droplet surface behaves mostly liquid-like during recalescence.

Summarising all our experimental insights and modelling, we can conclude that treating the droplet as a liquid for the purpose of calculating $F_a$ is reasonable, irrespective of nucleation position. Ice adhesion can be quantified via models such as the one by Meuler *et al.* but the adhesion values calculated coupled with our experimental observations support that, irrespective of the nucleation position, the droplet interface behaves in a predominantly liquid-like manner throughout recalescence.

Furthermore, in the ambient pressure experiments, the experimental outcome was independent of the nucleation position. Here, the recalescence force was reduced by up to six orders of magnitude, so that a change of the force of adhesion by one order of magnitude would not further affect the experimental outcome.

## Surface Tension

We are assessing the force acting on the droplet during the recalescence stage. It is therefore reasonable to employ the value of surface tension of water at 0 °C, which is $\sigma = 75.64$ mN m$^{-1}$ (Ref. [12]). $\sigma$ is a function of temperature. It is about 5% lower at room-temperature, and about 5% higher at -20 °C. While being substantial and important to e.g. high-precision contact angle measurements,[13] this uncertainty is within an acceptable range for the purposes of our scaling analysis.

## Capillary Pressure

For the micropillar surfaces, the capillary pressure resisting droplet impalement is computed as $p_{\mathrm{c}} = -(2f/(1-f))\cos\theta_{\mathrm{c}}(2\sigma/d)$, where $f = \pi d^2/4s^2$ is the wetting fraction.[14] To perform the same computation for the disordered spray-coated samples C1 and C2, we have to determine an effective pitch, $s$, diameter, $d$, and side-wall advancing contact angle, $\theta_{\mathrm{c}}$, for the surface structures.

In case of the glass coated with Coating A (sample C1), the underlying glass substrate is macroscopically smooth and all relevant roughness is added by the spray coating itself. To determine $s$ and $d$ for sample C1, we first obtained a linear height profile of the spray-coated surface using stylus profilometry (Bruker Dektak XT). Subsequently, we computed a linear best fit to the raw data and subtracted the result from the raw data to obtain a leveled height profile centered around the zero-height position, i.e. the average of the height profile is 0 (see Fig. S9). From contact angle measurements, we can conclude that the water droplets are resting on the spray-coated samples in a Cassie-Baxter wetting state, where they partially make contact with the spray coating while otherwise being suspended on air. As a first approximation, we propose that the water droplets make contact with all parts of the spray coating that are above the zero-height position, i.e. the hills, while the remaining parts of the surface, i.e. the valleys, remain filled with gas. Consequently, the droplet contact line is pinned at the height = 0 position, allowing us to compute an effective droplet-surface contact diameter of 32 µm, while the pitch between the hills is 74 µm, which can then be used to calculate $f$ and $p_{\mathrm{c}}$. From microscopy images of the C1 sample (Fig. S11), we see that the microscopic features created by Coating A are densely populated on the side-walls by the HFS nanoparticles that are a central ingredient of Coating A. Based on this observation, we assume the side-wall advancing contact angle of sample C1 to be $\theta_{\mathrm{c}} = 138°$, which is the advancing contact angle that we measured for a thin, conformal coating with the pure HFS-coating, i.e., Coating B (see Table S1).

In the case of the mesh coated with Coating A (sample C2), there are two potential impalement mechanisms that need to be considered: impalment into the spray-coating and impalement into the macroscopic pores of the mesh. Since the spray coating is the same as that used to make C1, we can state that the microtexture impalement $p_{\mathrm{c}}$ for both is the same. For impalement into the square lattice of the steel mesh, we must reformulate our calculation for wires with diameter $d_{\mathrm{m}}$ and centre-to-centre pitch $s_{\mathrm{m}}$ to be $p_{\mathrm{c}} = -(4f_{\mathrm{m}}/(1-f_{\mathrm{m}}))\cos\theta_{\mathrm{c}}(\sigma(s_{\mathrm{m}}-d_{\mathrm{m}})/d_{\mathrm{m}}(2s_{\mathrm{m}}-d_{\mathrm{m}}))$, where $f_{\mathrm{m}} = d_{\mathrm{m}}(2s_{\mathrm{m}}-d_{\mathrm{m}})/s_{\mathrm{m}}^2$ is the wetting fraction of the mesh and assuming $\theta_{\mathrm{c}} = 165°$, which is the advancing contact angle for Coating A. It is the impalement mechanism with the smaller value of $p_{\mathrm{c}}$ that sets the relevant resistance to impalement. For sample C2, it is the impalment into the macroscopic pores of the mesh that would be triggered first and is therefore limiting.

**Surface Property Effects**

In the main text, we demonstrate how changes in the surface geometry translate into variations in the forces acting on the droplet and how the balance of these forces, in turn, explains the observed icing phenomena. Here we want to make an attempt to provide an immediate link between surface geometry and icing phenomena.

We define $F_a$ as:

$$F_a = 2\pi R_c \sigma(1 + \cos\theta_r), \quad (9)$$

where (Ref. 15)

$$\theta_r = \lambda_p \theta_{r,flat} + (1 - \lambda_p)\theta_{air}, \quad (10)$$

with

$$\theta_{air} = 180°, \quad (11)$$

$$\lambda_p = \frac{1}{2}\left(\frac{d}{s}\right)\left[1 + \left(\frac{1}{2}\pi - 1\right)\left(\frac{d}{s}\right)\right]. \quad (12)$$

We define $F_c$ as:

$$F_c = \pi R_c^2 p_c (1 - f), \quad (13)$$

where (Ref. 14)

$$f = \frac{\pi}{4} d^2/s^2 \,, \quad (14)$$

$$p_c = -\left(\frac{2f}{1-f}\right)\cos\theta_c\left(\frac{2\sigma}{d}\right). \quad (15)$$

Note that the equation for $p_c$ is different in the case of the mesh surface (see SI, Capillary Pressure).

## Gravity

Gravity plays a minor role in determining the outcome of the freezing event. This can be understood by computing the Bond number, $Bo$, which scales gravity versus capillary forces and is defined as $Bo = \Delta\rho\ g\ L_c^2\ /\ \sigma$. Here, $\Delta\rho$ is the density difference between the liquid droplet and the gaseous environment. $\Delta\rho$ can be approximated as the density of the liquid, $\Delta\rho \approx \rho_L$. $g$ = 9.81 m $s^{-2}$ is the gravitational acceleration, $L_c$ is the characteristic length, for a droplet typically set to the droplet radius $L_c = R_d$, and $\sigma$ is the surface tension. For a 10 μL water droplet as studied here, $Bo \approx 0.2$. From this we see that gravity has a minor effect on the droplet, whose shape and deformation are dominated by surface tension. While the droplet is resting on the pillars, the gravitational force acting on it is always compensated by equal but opposing forces exerted on it through the pillars. Therefore, during impalement and suffusion, the gravitational force does not affect the outcome. For the case of expulsion, the gravitational force does not determine if expulsion occurs or not. As we observe experimentally (see Fig. 1 and Movie S2), the centre of gravity of a droplet hardly lifts upwards during the initial stages of expulsion. The adhesive forces between the pillars and the droplet dominate the resisting force to expulsion. Once the droplet is completely released from the substrate, gravity comes into play and limits the overall height of the expulsion event. In summary, it is valid to not consider gravitational forces within our force analysis.

## Substrate Thermal Conductivity and Elastic Modulus

To investigate the effect of substrate thermal conductivity and elastic modulus, we have performed freezing experiments on silicon micropillar surfaces (samples S1s, S2s, S3s and S4s; n = 10 experiments each; see Table S1 for details), which are geometrically equivalent to some of the PDMS substrates (samples S1, S2, S3 and S4). Both thermal conductivity and elastic modulus are more than four orders of magnitude higher in silicon as compared to PDMS. Whilst for silicon thermal conductivity and elastic modulus are on the order of 100 W $m^{-1}$ $K^{-1}$ and 100 GPa, respectively, for PDMS, they are on the order of 0.1 W $m^{-1}$ $K^{-1}$ and 1 MPa (depending on the degree of crosslinking), respectively. Fig. S13 shows that silicon substrates demonstrate similar behaviour to PDMS concerning impalement and expulsion at low pillar diameter, $d$. In the limit of high $d$, PDMS samples showed suffusion, while silicon samples did not. Instead, at $d$ = 39 µm on the S1s silicon sample, 7 out of 10 droplets did not freeze, but spontaneously oscillated vertically before laterally translating out of the field of view. The lack of suffusion on silicon can be partly attributed to the higher receding contact angles of the silicon samples.

## Surfactant Effect

We have performed experiments with droplets that contain specific concentrations of sodium dodecyl sulphate (SDS), a surfactant. Adding a surfactant to water is an effective way to change the surface tension of the liquid. By adding SDS in different concentrations (0.3, 1, and 3 mmol $L^{-1}$), we have reduced the surface tension from the value of pure water at 0 °C ($\sigma$ = 75.64 mN $m^{-1}$) to values of 71.47, 68.49, and 57.28 mN $m^{-1}$, respectively.[16] As shown in Fig. S14, we have studied samples S1, D1, D3, D4 and D5.

First, we studied how the addition of SDS can affect the measured contact angles (Fig. S14a,b). For most samples, adding SDS to the water does not substantially affect the advancing contact angle. As shown in Fig S14a, the experimental spread of the measured advancing contact angles for a given sample and SDS concentration (represented by the error bars) can often overlap amongst all four SDS concentrations. This implies that the advancing contact angle of the evaluated surfaces is comparably stable against the addition of SDS. In contrast, for some samples, adding SDS reduces the receding contact angle, as shown in Fig. S14b. This effect is also observed on flat, untextured PDMS (Fig. S14c). We note that this effect serves to increase the contact radius, $R_c$, of the droplet due to the reduction in the static contact angle.

In Fig. S14d, we show the experimental outcomes. On sample S1, we did not observe an effect of adding SDS. The occurrence of suffusion remained at 100%. For samples D1, D3, and D4, we observed an increase in suffusion events as a consequence of adding SDS, while impalement was not enhanced. For sample D5, we found that adding SDS might even reduce the occurrence of impalement.

To understand some of these findings, we have computed the governing force ratios ($F_c$ / $F_r$) and ($F_r$ / $F_a$) in Fig. S14e,f. While seemingly one should expect the ratio of ($F_r$ / $F_a$) to increase with adding surfactant—due to a decrease in surface tension—adding surfactant also causes other important variables to change, namely, the apparent contact angle and the contact radius. Whilst adding SDS reduces $\sigma$, it actually increases $R_c$ and ($1 + \cos\theta_r$) somewhat. Further, it is important to note that adding SDS also affects the saturated vapor pressure above the aqueous solution. For small amounts of SDS (as studied here), the saturated vapor pressure decreases with the addition of SDS.[17] Based on the literature data, we estimate the reduction in vapor pressure to be at least 0.6%, 2.1%, and 6.3% due to the addition of 0.3, 1, and 3 mmol $L^{-1}$, respectively. Since the recalescence force, $F_r$, scales with the square of the vapor pressure, this can result in a reduction of $F_r$ of 12%. This represents a conservative estimate as the concentration will increase as the droplet evaporates, especially at the liquid-vapour interface due to the slow diffusion of SDS in water when compared to the timescale of an experiment.[18] Both force ratios are therefore reduced for increasing SDS, as demonstrated in Fig. S14g,h in which increasing the concentration of SDS ten-fold changes the most probable outcome for a D3 substrate from expulsion to suffusion.

For sample S1, adding SDS shows no effect, since irrespective of SDS concentration ($F_c$ / $F_r$) >> 1 and ($F_r$ / $F_a$) < 1, preventing impalement and favouring suffusion over expulsion, respectively.

For sample D1, adding SDS reduces the occurrence of expulsion. While for pure water both expulsion and suffusion were observed, all experiments for droplets containing surfactant showed suffusion. Not observing any impalement on sample D1—irrespective of SDS concentration—is expected since ($F_c$ / $F_r$) >> 1 for all tested SDS concentrations. At the same time, for sample D1, ($F_r$ / $F_a$) remains close to unity—irrespective of SDS concentration. This renders both expulsion and suffusion the expected experimental outcomes.

For sample D3, adding SDS increases the occurrence of suffusion. Irrespective of SDS concentration, the ratio ($F_c$ / $F_r$) >> 1, which prevents impalement. However, as the SDS concentration increases, ($F_r$ / $F_a$) monotonically approaches unity, thereby explaining the increase of suffusion.

In a similar manner, for sample D4, adding SDS increases the occurrence of suffusion. Irrespective of SDS concentration, the ratio ($F_c$ / $F_r$) > 1, making impalement less likely. However, as the SDS concentration increases, again, ($F_r$ / $F_a$) is reduced below the value of 2, thereby explaining the occasional occurrence of suffusion.

For sample D5, irrespective of SDS concentration, expulsion is the dominant outcome. While irrespective of SDS concentration ($F_r$ / $F_a$) >> 1 prevents the occurrence of suffusion, adding SDS slightly increases ($F_c$ / $F_r$), thereby rationalizing the reduced occurrence of impalement events observed for higher SDS concentrations.

## Droplet Size

In order to quantify the effect of droplet size on the observed outcome, we conducted a series of experiments with droplet volumes differing from the 10 μL used throughout the remainder of the study. In the lower limit, a minimum volume of 2 μL was set due to the challenges of reliably placing smaller droplets on most surfaces. Furthermore, the contact radii of small droplets approach the interpillar pitch, exposing other mechanisms for impalement transitions which are not within the scope of this study. In the upper limit, a maximum volume of 20 μL was chosen to avoid gravitational effects which deform large droplets.

Experiments in the range 2 μL ≤ droplet volume ≤ 20 μL are depicted in Fig. S16. Across the substrates tested (S1, D1, D3 and D5), we found no major effect of the droplet volume on the observed outcome suggesting that the phenomena are largely independent of the droplet size. This result agrees well with our modelling in which the three principal forces of $F_r$, $F_a$ and $F_c$ scale proportionally to $R_d^2$, $R_d$ and $R_d^2$, respectively. Therefore, the ratio ($F_c$ / $F_r$) should be independent of $R_d$ and ($F_r$ / $F_a$) ~ $R_d$. Noting that an order of magnitude change in droplet volume corresponds to only a factor of two change in $R_d$, both force ratios are, consequently, largely insensitive to variations in droplet size.

## Thermal Profile of a Freezing Droplet

The temperature increase of a droplet during recalescence from a state of supercooled liquid ($T_d \ll 0°C$) before nucleation to the equilibrium freezing temperature ($T_d = 0°C$) afterwards is well documented.[8,19] However, the subsequent temperature history depends on the nature of the second, crystallisation, stage of freezing. To determine this, we inserted thermocouples (Type T (Thickness 0.0787 mm), Omega) into droplets and performed experiments according to the standard protocols. It should be noted that for the low-pressure experiments, freezing was unaffected by the insertion of the thermocouple as the free surface remained favourable for nucleation. However, the thermocouple provided a site for ice nucleation in some of the ambient-pressure experiments due to the hydrophilic nature of the metal sensor. This increases the droplet temperature when freezing is initiated but has no marked effect on the post-recalescence behaviour.

The time evolution of droplet temperature during freezing experiments performed at low-pressure can be seen in Fig. S18a. Recalescence can be observed as a sharp rise in the droplet temperature with $T_d \approx 0°C$ achieved in all cases irrespective of the degree of supercooling as expected. After recalescence, and despite the change of phase, evaporation from the surface in the low-pressure environment remains high, resulting in the droplet rapidly cooling again over the course of a handful more milliseconds. Contrastingly in the ambient-pressure freezing case (Fig. S18b), the rate of cooling post-recalescence is substantially slower resulting in the droplet remaining warmer than the ambient environment for a significant amount of time. This can be attributed to the primary method of cooling being conduction to the environment as evaporation is heavily reduced when compared to the low-pressure case.

Whilst recalescence is an adiabatic process in which the released latent heat increases the droplet temperature to the equilibrium freezing temperature, crystallisation is an isothermal process limited by the rate at which latent heat can diffuse from the droplet into the environment. Therefore, crystallisation maintains the droplet at the equilibrium freezing temperature until the droplet is fully solidified. From the freezing temperature profiles plotted in Fig. S18, we can see that under vacuum conditions, the droplet rapidly cools again post-recalescence, signifying that the crystallisation stage is rapid. This is to be expected as, in a low-pressure environment, the partially frozen droplet is still able to strongly evaporate and remove latent heat through this mechanism. Contrastingly, for the low-temperature, ambient-pressure case, we observe that the droplet temperature remains close to the equilibrium freezing temperature for a much longer time as evaporation is no longer the dominant mechanism for latent heat release. Thus, post-recalescence phenomena are observed over a longer timescale in the low-temperature, ambient-pressure case.

In the low-pressure case, the droplet remains colder than the environment throughout the freezing process meaning that condensation and freezing within the texture is not possible. Volumetric expansion of the droplet during crystallisation does, however, lead to suffusion of the droplet core into the texture as the droplet solidifies from the free surface inwards due to the aforementioned heat removal via evaporation.

In the low-temperature, ambient-pressure case, the droplet is initially in thermal equilibrium with the surrounds. However, post-recalescence and throughout the crystallisation stage, the

droplet is significantly warmer than the surroundings. This results in a local supersaturation of the environment close to the slowly evaporating droplet which facilitates the condensation, coalescence and bottom-up suffusion illustrated in Fig. 4 and Movie S6. From the side-view imaging, it is apparent that crystallisation initiates at the base of the droplet (as seen from the dissolution of gas in the form of entrapped bubbles post-crystallisation), suggesting that conduction to the substrate is the dominant mechanism of heat removal. Some volumetric expansion is also observed in this case with a bulge typically emerging from the droplet free surface as can be discerned in Fig. 4a panels 5 and 6.

## Supplementary references


1. Jung, S., Tiwari, M. K. & Poulikakos, D. Frost halos from supercooled water droplets. *Proc. Natl. Acad. Sci.* **109**, 16073–16078 (2012).
2. Graeber, G., Dolder, V., Schutzius, T. M. & Poulikakos, D. Cascade Freezing of Supercooled Water Droplet Collectives. *ACS Nano* **12**, 11274–11281 (2018).
3. Lu, Z., Kinefuchi, I., Wilke, K. L., Vaartstra, G. & Wang, E. N. A unified relationship for evaporation kinetics at low Mach numbers. *Nat. Commun.* **10**, 2368 (2019).
4. Vaartstra, G., Lu, Z., Lienhard, J. H. & Wang, E. N. Revisiting the Schrage Equation for Kinetically Limited Evaporation and Condensation. *J. Heat Transfer* **144**, (2022).
5. Smith, J. D., Cappa, C. D., Drisdell, W. S., Cohen, R. C. & Saykally, R. J. Raman thermometry measurements of free evaporation from liquid water droplets. *J. Am. Chem. Soc.* **128**, 12892–12898 (2006).
6. Pruppacher, H. R. & Klett, J. D. *Microphysics of Clouds and Precipitation*. (Springer, 2010). doi:10.1007/978-0-306-48100-0.
7. Graeber, G., Schutzius, T. M., Eghlidi, H. & Poulikakos, D. Spontaneous self-dislodging of freezing water droplets and the role of wettability. *Proc. Natl. Acad. Sci.* **114**, 11040–11045 (2017).
8. Jung, S., Tiwari, M. K., Doan, N. V. & Poulikakos, D. Mechanism of supercooled droplet freezing on surfaces. *Nat. Commun.* **3**, 615 (2012).
9. Gao, L. & McCarthy, T. J. Teflon is Hydrophilic. Comments on Definitions of Hydrophobic, Shear versus Tensile Hydrophobicity, and Wettability Characterization. *Langmuir* **24**, 9183–9188 (2008).
10. Butt, H.-J. *et al.* Energy Dissipation of Moving Drops on Superhydrophobic and Superoleophobic Surfaces. *Langmuir* **33**, 107–116 (2017).
11. Meuler, A. J. *et al.* Relationships between Water Wettability and Ice Adhesion. *ACS Appl. Mater. Interfaces* **2**, 3100–3110 (2010).
12. Vargaftik, N. B., Volkov, B. N. & Voljak, L. D. International Tables of the Surface Tension of Water. *J. Phys. Chem. Ref. Data* **12**, 817–820 (1983).
13. Chulkova, E. V., Emelyanenko, K. A., Emelyanenko, A. M. & Boinovich, L. B. Elimination of wetting study flaws in unsaturated vapors based on Laplace fit parameters. *Surf. Innov.* **10**, 21–24 (2022).
14. Bartolo, D. *et al.* Bouncing or sticky droplets: Impalement transitions on superhydrophobic micropatterned surfaces. *Europhys. Lett.* **74**, 299–305 (2006).
15. Extrand, C. W. Model for Contact Angles and Hysteresis on Rough and Ultraphobic Surfaces. *Langmuir* **18**, 7991–7999 (2002).

16. Wang, X., Chen, L. & Bonaccurso, E. Comparison of spontaneous wetting and drop impact dynamics of aqueous surfactant solutions on hydrophobic polypropylene surfaces: scaling of the contact radius. *Colloid Polym. Sci.* **293**, 257–265 (2014).
17. Li, P., Han, B., Yan, H. & Liu, R. Vapor Pressure of the Aqueous Solution of Sodium Dodecyl Sulfate. *J. Chem. Eng. Data* **41**, 285–286 (1996).
18. Winkelmann, J. Diffusion coefficient of sodium dodecyl sulfate in water. in *Diffusion in Gases, Liquids and Electrolytes* 1478–1481 (Springer Berlin Heidelberg, 2018). doi:10.1007/978-3-662-54089-3_998.
19. Jung, S. *et al.* Are Superhydrophobic Surfaces Best for Icephobicity? *Langmuir* **27**, 3059–3066 (2011).
20. Mysels, K. J. Surface tension of solutions of pure sodium dodecyl sulfate. *Langmuir* **2**, 423–428 (1986).
21. Wei, J. *et al.* Enhanced thermal conductivity of polydimethylsiloxane composites with carbon fiber. *Compos. Commun.* **17**, 141–146 (2020).
22. Wang, Z., Volinsky, A. A. & Gallant, N. D. Crosslinking effect on polydimethylsiloxane elastic modulus measured by custom-built compression instrument. *J. Appl. Polym. Sci.* **131**, n/a-n/a (2014).

# Supplementary Figures

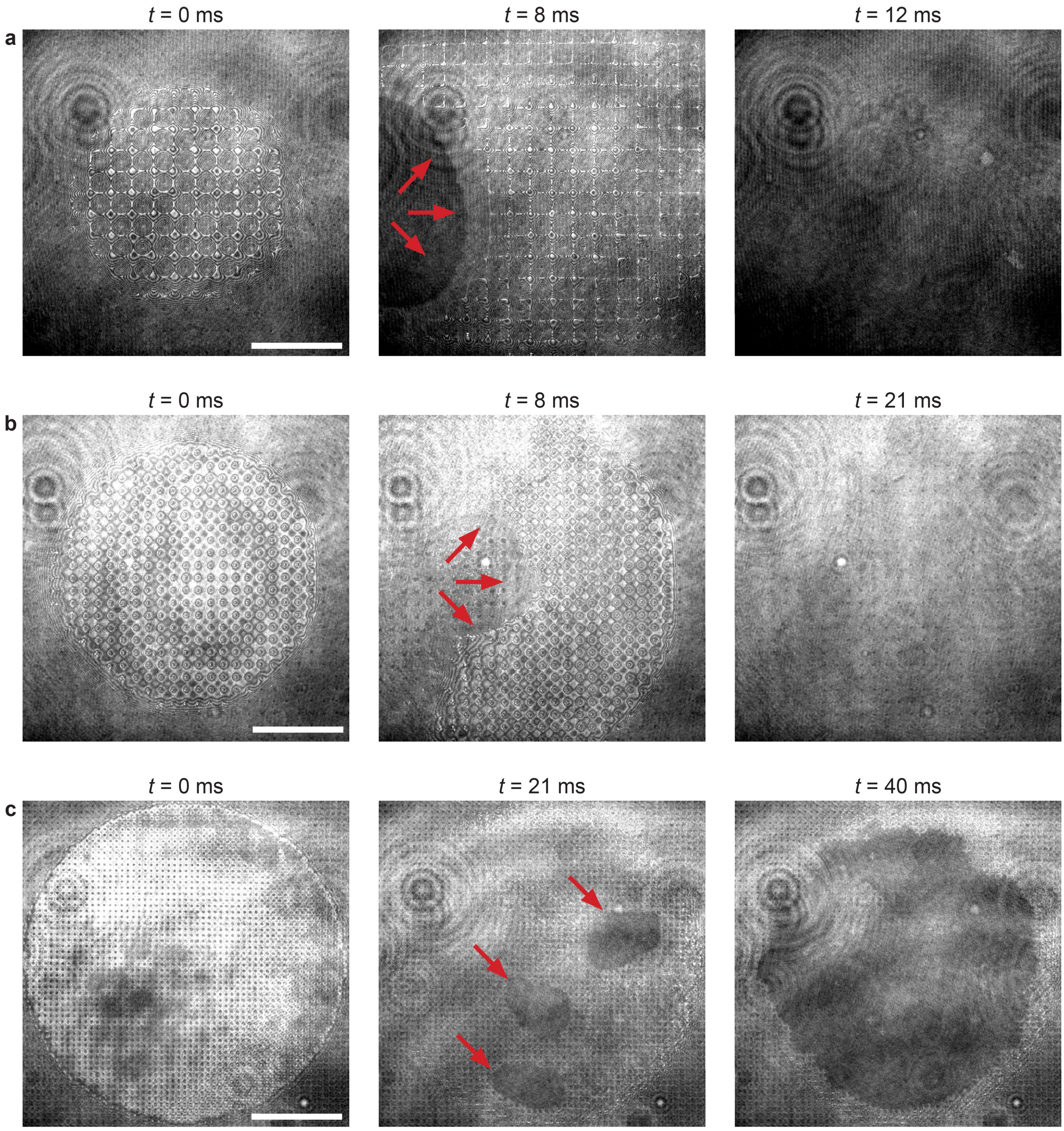


**Fig. S1. Enlarged bottom-view images of key events of the observed phenomena**. Snapshots for impalement (**a**), expulsion (**b**), and suffusion (**c**). Red arrows highlight impalement (**a**), de-wetting (**b**), and the onset of suffusion (**c**). See caption of Fig. 1 for further details.

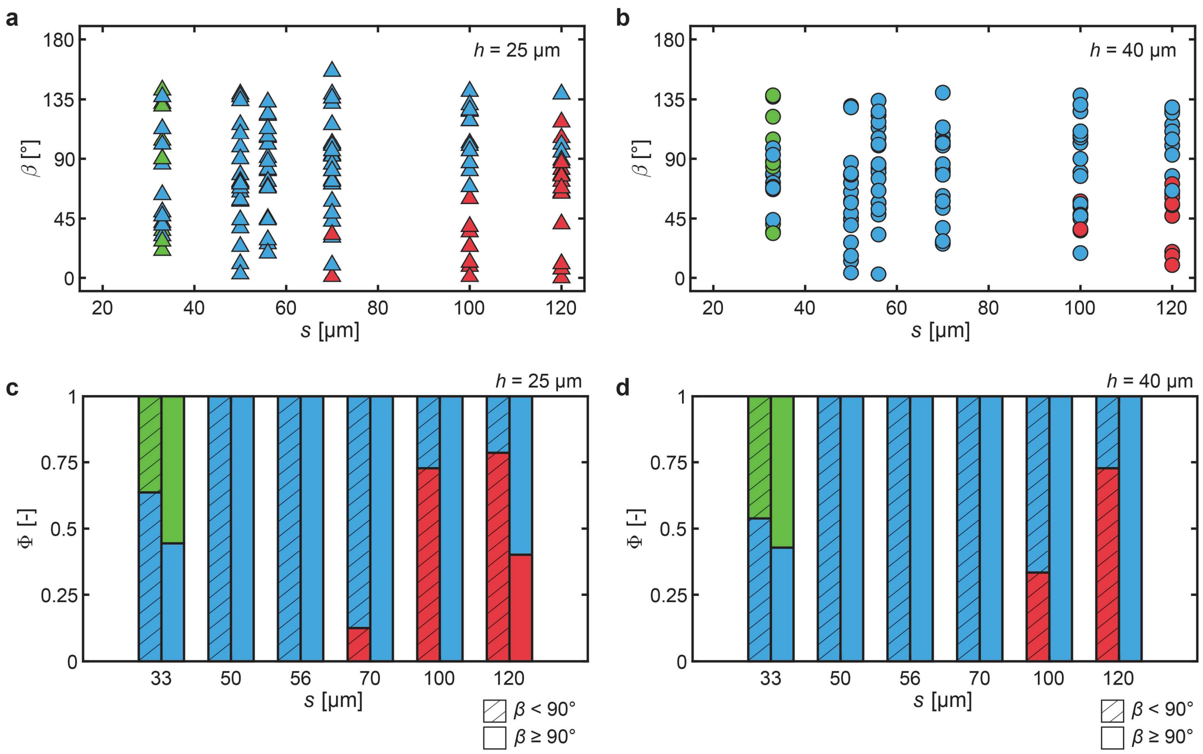


**Fig. S2. Scatter plots and height distinction of all individual N = 249 experiments performed for Fig. 2.** (**a**) Outcomes observed *vs*. pillar pitch, *s*, and nucleation angle, *β*, for a pillar height of *h* = 25 μm (red, impalement; blue, expulsion; green, suffusion). (**b**) Outcomes observed *vs*. *s* and *β* for *h* = 40 μm. (**c**) Bar chart of Φ for each *s* as a function of nucleation angle *β*, for a pillar height of *h* = 25 μm (hashed bar, $\beta < 90°$; plain bar, $\beta \geq 90°$). (**d**) Bar chart of Φ for each *s* as a function of nucleation angle *β*, for a pillar height of *h* = 40 μm (hashed bar, $\beta < 90°$; plain bar, $\beta \geq 90°$). Employed surfaces: D1 to D6 – see Table S1 for details. Note that even on the same employed surface with identical *β*, two different outcomes can be observed. This highlights the uncertainty induced by the inherently stochastic nature of the freezing process and, more specifically, nucleation itself. The freezing delay (time between opening the valve towards the vacuum pump and droplet nucleation) is distributed over a few seconds for individual experiment. As a result, droplets experience different degrees of supercooling at the moment of nucleation, which translates into a difference in the recalescence force (see SI, Vapour Flux).

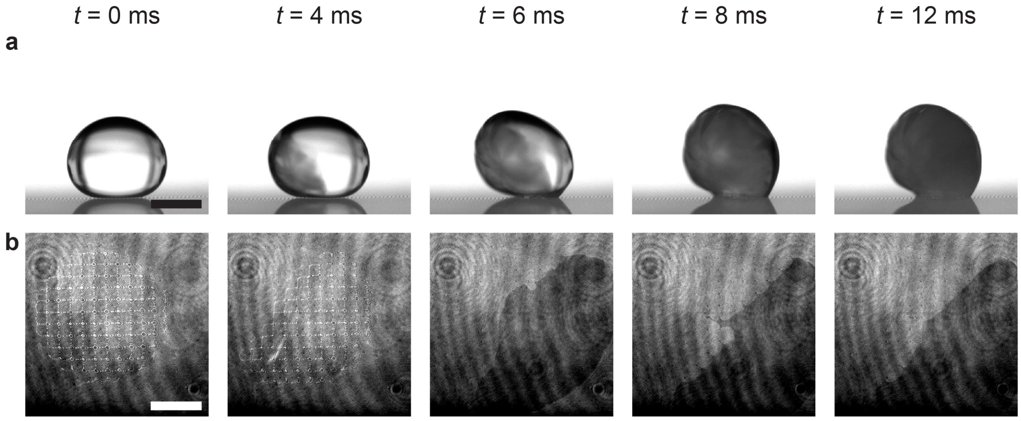


**Fig. S3. Rolling impalement.** Synchronised (**a**) side- and (**b**) bottom-view image sequences of the impalement mechanism for a nucleation angle $\beta \geq 90°$, i.e., nucleation below the centreline of the droplet. The contact line on the non-frozen side is pinned leading to rotation of the droplet, redirecting the recalescence force into the texture. (Scale bars: **a,** 2 mm; **b**, 500 μm.)

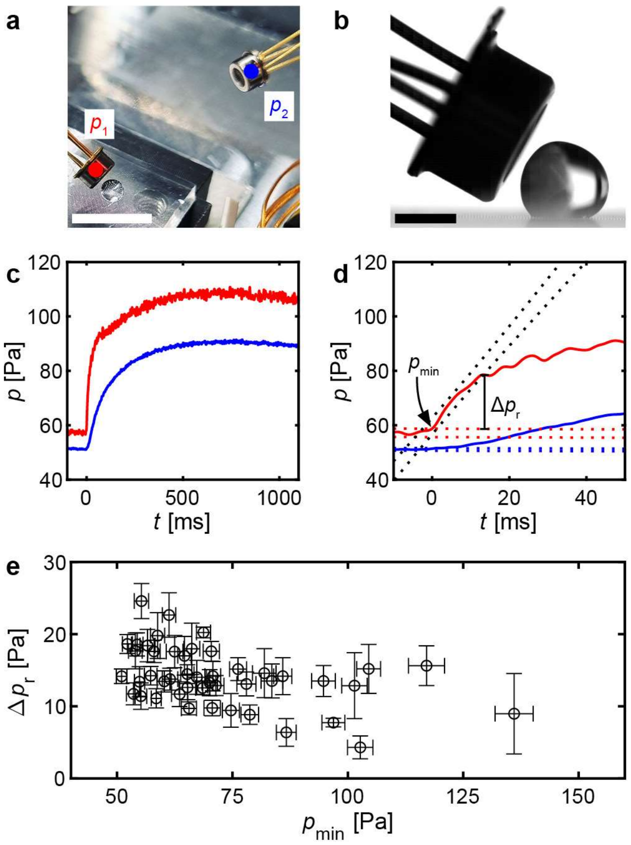


**Fig. S4. Quantifying the explosive vapour release due to recalescence.** (**a**) Experimental setup to measure the released vapour pressure. (Distance to sensors: $p_1$, < 1 mm; $p_2$, ~ 20 mm). (**b**) Magnified view of $p_1$ sensor during recalescence. (**c**) Typical pressure responses over a freezing event for the $p_1$ and $p_2$ sensors (red and blue lines, respectively). (**d**) Magnified view of a typical pressure response focussing on the recalescence stage of freezing. The dashed red and blue lines represent corridors of 99.95% confidence around the mean of the pressure trend before nucleation used to calculate the minimum pressures reached, $p_{min}$. We define $t = 0$ as the time at which the $p_1$ sensor leaves its corridor, indicating that recalescence has started. The dashed black line represents a corridor of 99.95% confidence around the iterative, linear fit of $p_1$ after nucleation. The pressure at which the linear fit exits the corridor is compared to $p_{min}$ to calculate the pressure increase near the droplet over recalescence, $\Delta p_r$. (**e**) $\Delta p_r$ *vs*. $p_{min}$ for all experiments (N = 46) showing an average and standard deviation of 14 ± 4 Pa. The placement of the sensor did not affect droplet nucleation and recalescence. (Scale bars: **a**, 10 mm; **b**, 1 mm.)

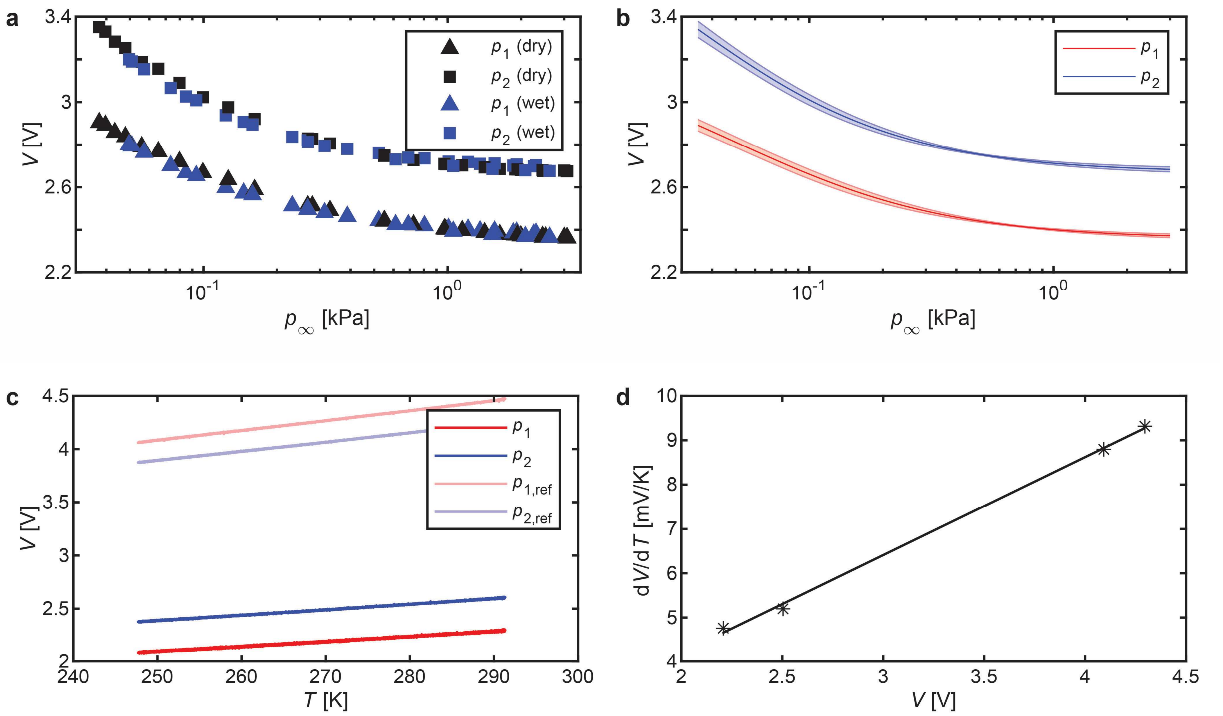


**Fig. S5. Pirani pressure sensor calibration.** (**a**) Plot of voltage *vs*. pressure for both sensors in low- and high-water vapour concentration atmospheres. (**b**) Resulting calibration curves accounting for unknown gas compositions. (**c**) Plot of voltage *vs*. temperature for both sensors and their sealed reference counterparts. (**d**) Plot of voltage temperature drift *vs*. voltage at a temperature of 273.15 K for each sensor. The gradient of the best fit line equals the constant temperature coefficient of resistance, $C_T$.

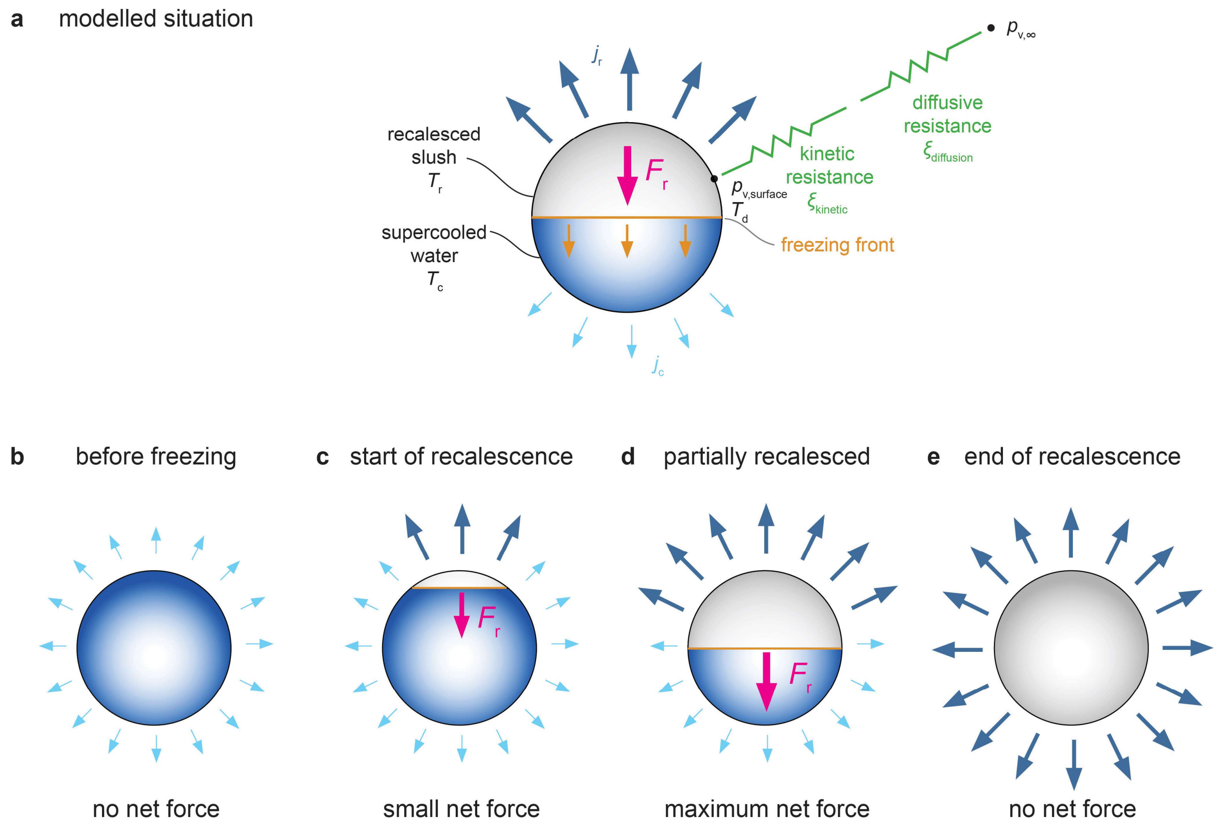


**Fig. S6. Modelling droplet evaporation and the recalescence force.** (**a**) Spherical droplet undergoing recalescence from an initially supercooled state. At the moment of evaluation, the recalescence freezing front has progressed through half of the droplet. Behind the freezing front, the droplet is slush (mixture of water and ice) at the equilibrium freezing temperature, $T_r = 0$ °C, while in front of the freezing front, the droplet is still a supercooled liquid at a temperature, $T_c$. The evaporation rate from the upper slush hemisphere, $j_r$, is substantially higher than the evaporation rate from the bottom supercooled hemisphere, $j_c$. The evaporation rates can be computed based on the kinetic and diffusive evaporation resistances, $\xi_{kinetic}$ and $\xi_{diffusion}$, and the driving vapour pressure difference between the vapour pressure at the surface, $p_{v,surface}$, and the vapor pressure at a distance from the droplet, $p_{v,\infty}$. (**b-e**) Schematic representations of the droplet (b) before, (c) at the start, (d) in the middle and (e) after completion of recalescence, respectively.

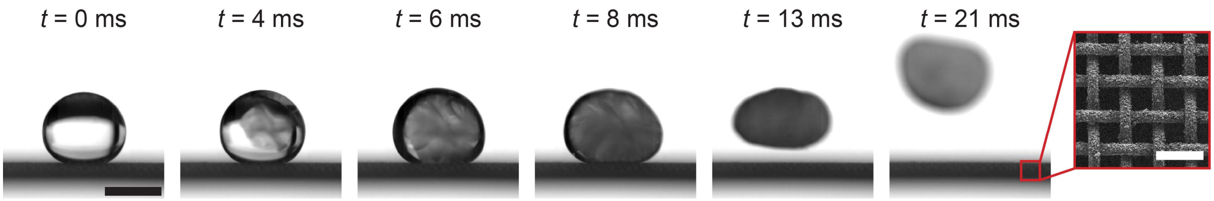


**Fig. S7. Expulsion on porous substrates.** High-speed image sequences of recalescence on a superhydrophobic steel mesh (sample C2, see Table S1 for details). Expulsion is observed despite the possibility for vapour to drain through the texture with the implication that an overpressure is not required for ice expulsion. (Scale bars: 2 mm; Inset, 400 μm.)

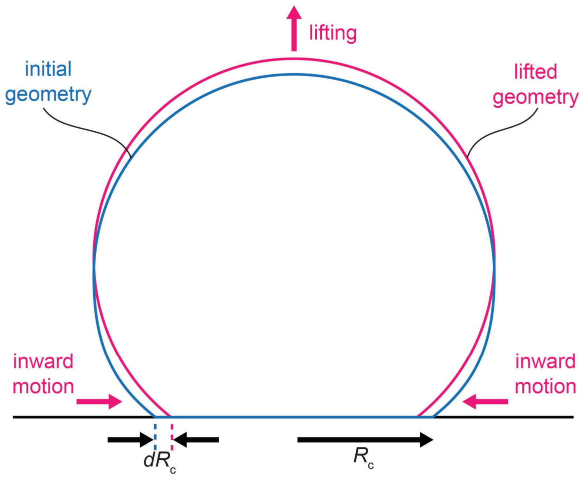


**Fig. S8. Schematic illustrating the derivation of the adhesion force.** Lifting of a sessile droplet causes the droplet triple line of length $2\pi\ R_c$ to radially recede by a distance $dR_c$.

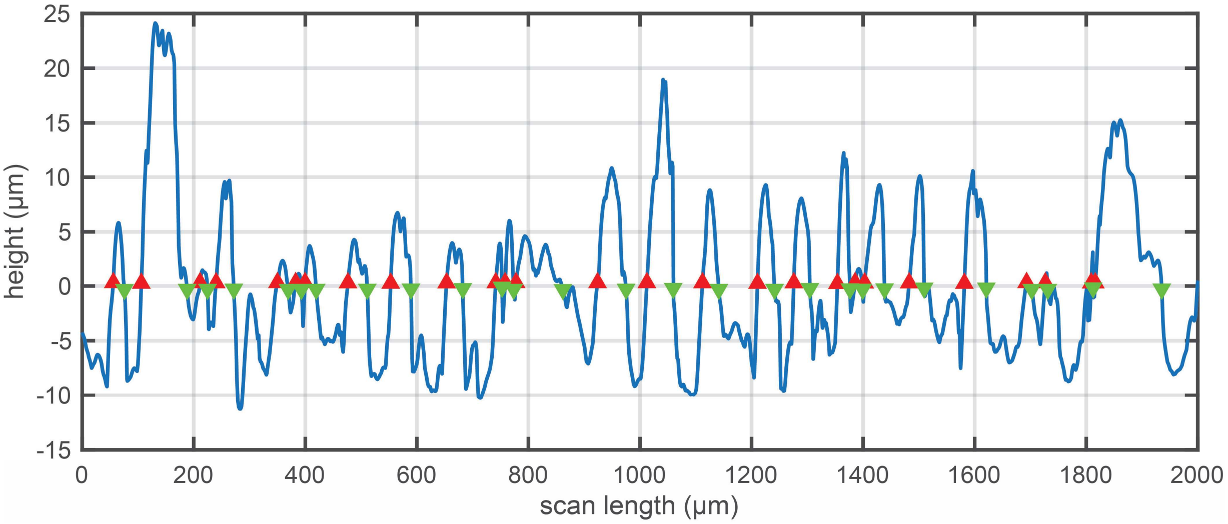


**Fig. S9. Height profile of glass sample coated with Coating A (identifier C1) as obtained by profilometry.** The blue line shows the leveled height data. Red upward pointing triangles highlight where the height profile crosses the height = 0 line with a positive slope. Green downward pointing triangles show where the height profile crosses the height = 0 line with a negative slope.

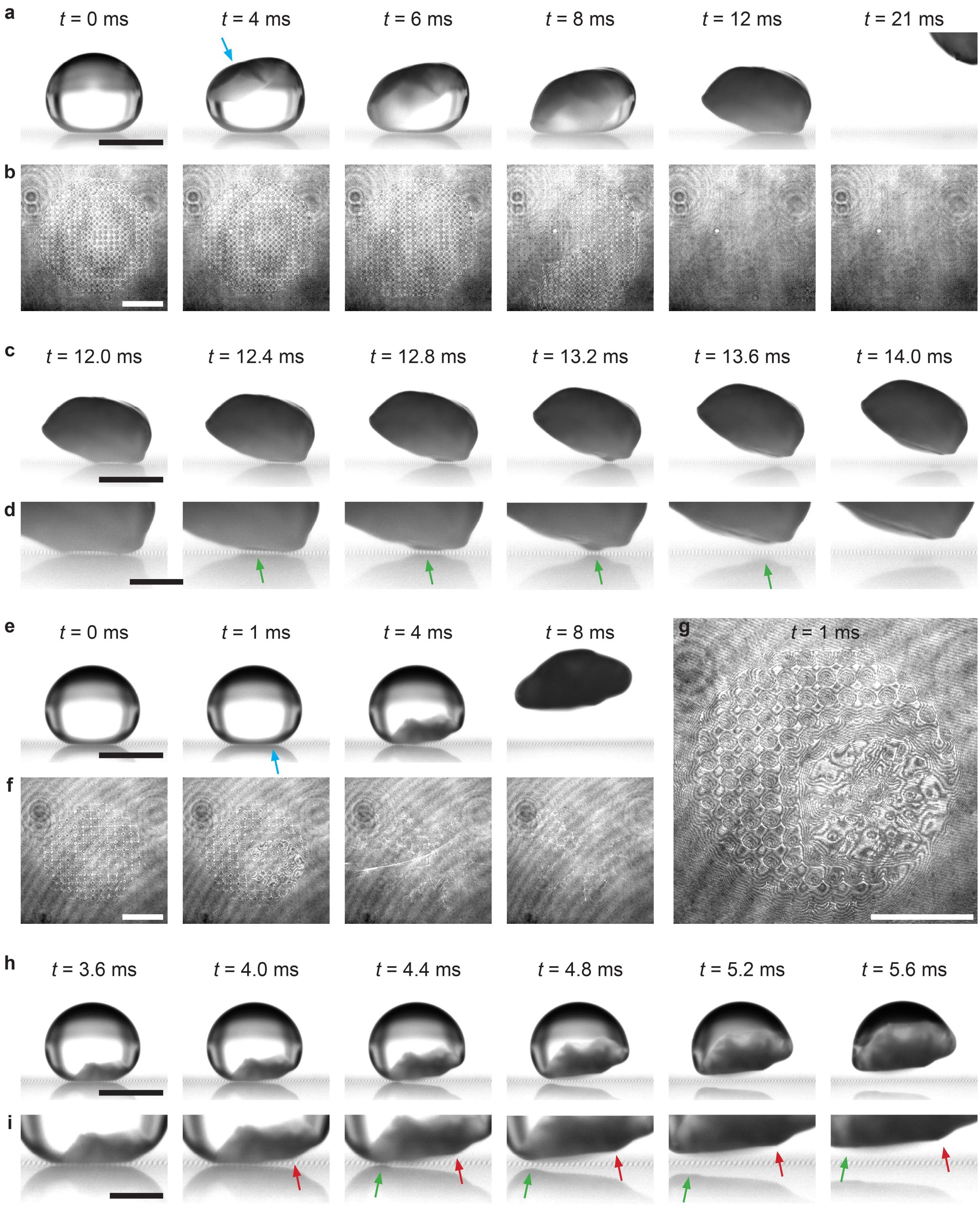


**Fig. S10. Dynamics of the droplet interface close to the substrate during an expulsion event as a function of nucleation position. a – d**, Homogeneous droplet nucleation from the droplet/air interface (indicated by the blue arrow in **a**). Synchronised (**a**) side- and (**b**) bottom-view of the expulsion event. (**c**) Image sequence focussing on the moment of droplet departure from the

substrate with (**d**) magnified side-views showing the droplet interface near the substrate. The droplet/substrate interface deforms during and after expulsion, suggesting a liquid-like behaviour throughout the droplet detachment process (green arrows in **d**). **e** – **i**, Heterogeneous droplet nucleation from the droplet/substrate interface (indicated by a blue arrow in **e**). Synchronised (**e**) side- and (**f**) bottom-view of the expulsion event. (**g**) Magnified bottom-view image showing that interference patterns remain during the expulsion event — an indication that the liquid-air interface has not yet solidified. Once the interface has partially solidified, the incident light is scattered, and the contrast is lost. (**h**) Image sequence focussing on the moment of droplet departure from the substrate with (**i**) magnified side-views showing two distinct behaviours of the droplet interface near the substrate. Whilst close to the nucleation point, the interface preserves its shape after expulsion, suggesting a rather solid-like behaviour (red arrows in **i**), away from the point of nucleation, the interface deforms during and after expulsion, suggesting a more liquid behaviour (green arrows in **i**). (Scale bars: **a, c, e, h,** 2 mm; **b, f, g,** 500 μm; **d, i,** 1 mm. Employed surfaces: **a** – **d**, D4, **e** – **i**, D5 (see Table S1 for details).

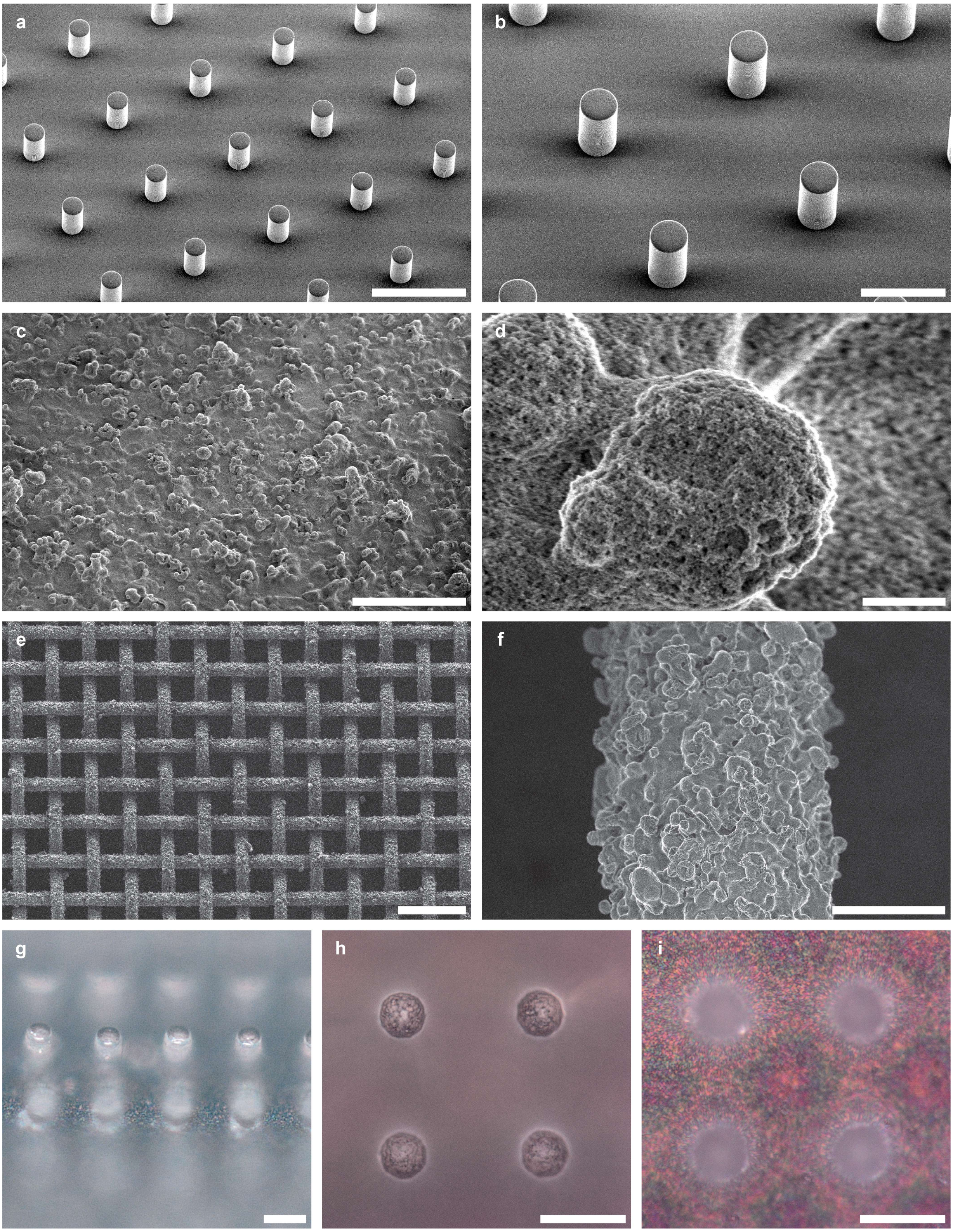


**Fig. S11. Scanning electron micrographs and optical images of the substrates used in this study. (a,b),** PDMS micropillar surface (S3), (**c,d**), Glass coated with Coating A (C1), (**e,f**), Metal mesh coated with Coating A (C2), and (**g,h,i**), PDMS micropillar surface coated with Coating B

(D1*) focussing on the side, at the top and at the bottom of the pillars, respectively. (Scale bars: **a,** 100 μm; **b,** 50 μm; **c,** 100 μm; **d,** 2 μm; **e,** 500 μm; **f,** 50 μm; **g,h,i,** 20 μm.)

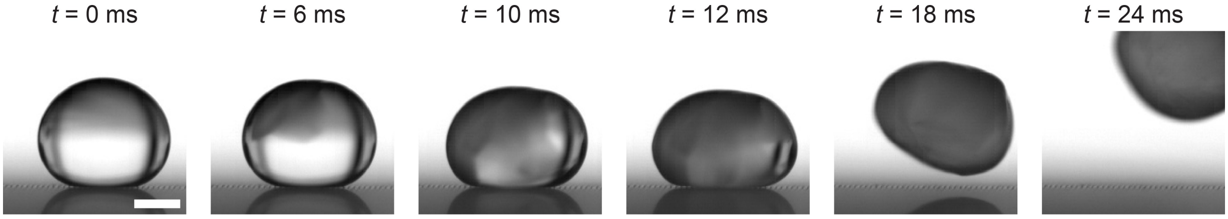


**Fig. S12. Expulsion event after temporary transition into macro-Wenzel state.** High-speed image sequence of recalescence in a low-pressure environment on a superhydrophobic, HFS-coated PDMS micropillar sample (D6*, see Table S1 for details). Expulsion is observed despite a temporary transition into a micro-Wenzel wetting state, highlighting that a hydrophobic coating can effectively prevent impalement events by maintaining a nano-Cassie state. (Scale bar: 1 mm.)

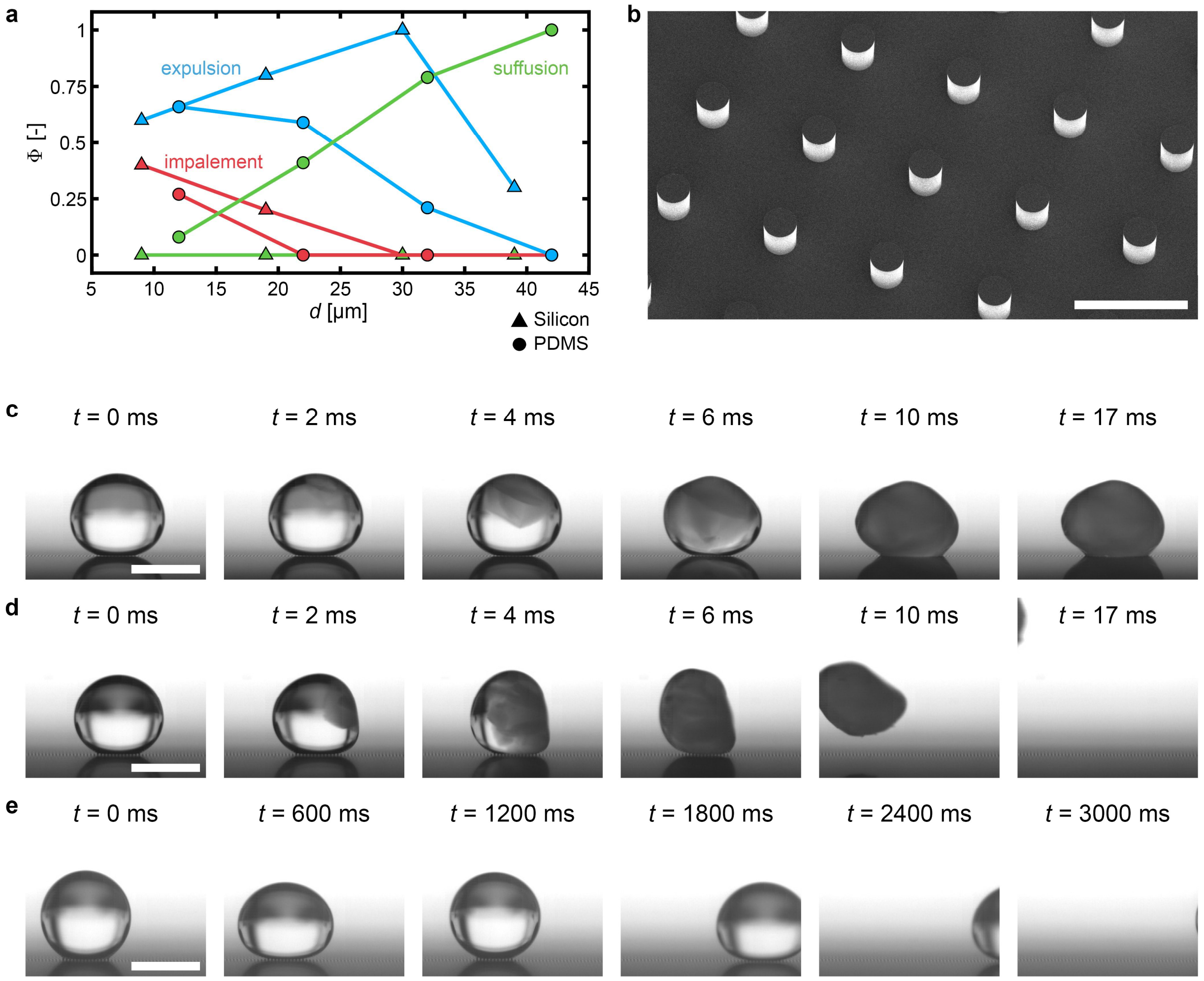


**Fig. S13. Effect of substrate thermal conductivity and rigidity on freezing-induced wetting transitions.** **a**, Experimental outcome probability of low-pressure droplet freezing events on micropillar substrates as a function of pillar diameter, *d*, and substrate material (FDTS-treated silicon versus PDMS), while maintaining a constant pillar pitch of $s$ = 100 µm. Outcomes are differentiated by colour (red, impalement; blue, expulsion; green, suffusion). Triangles represent the silicon micropillar substrates (samples S1s, S2s, S3s and S4s; n = 10 experiments each), while circles represent geometrically equivalent PDMS micropillar substrates (sample S1, S2, S3 and S4; n ≥ 39 each) – see Table S1 and S2 for details. At $d$ = 39 µm on the S1s silicon sample, 7 out of 10 droplets did not freeze, but spontaneously oscillated vertically before laterally translating out of the field of view. **b**, Scanning electron microscopy image of an S2s sample. **c**, Droplet impalement observed on an S4s sample showing a thin intervening liquid layer between the freezing droplet and the substrate. **d**, Droplet expulsion observed on an S2s sample. **e**, Spontaneous droplet oscillations observed on an S1s sample. See Table S1 for details about the samples. Initial droplet volume, $V$ = 10 µL. Scale bars: **b**, 100 µm; **c**, **d**, **e**, 2 mm.

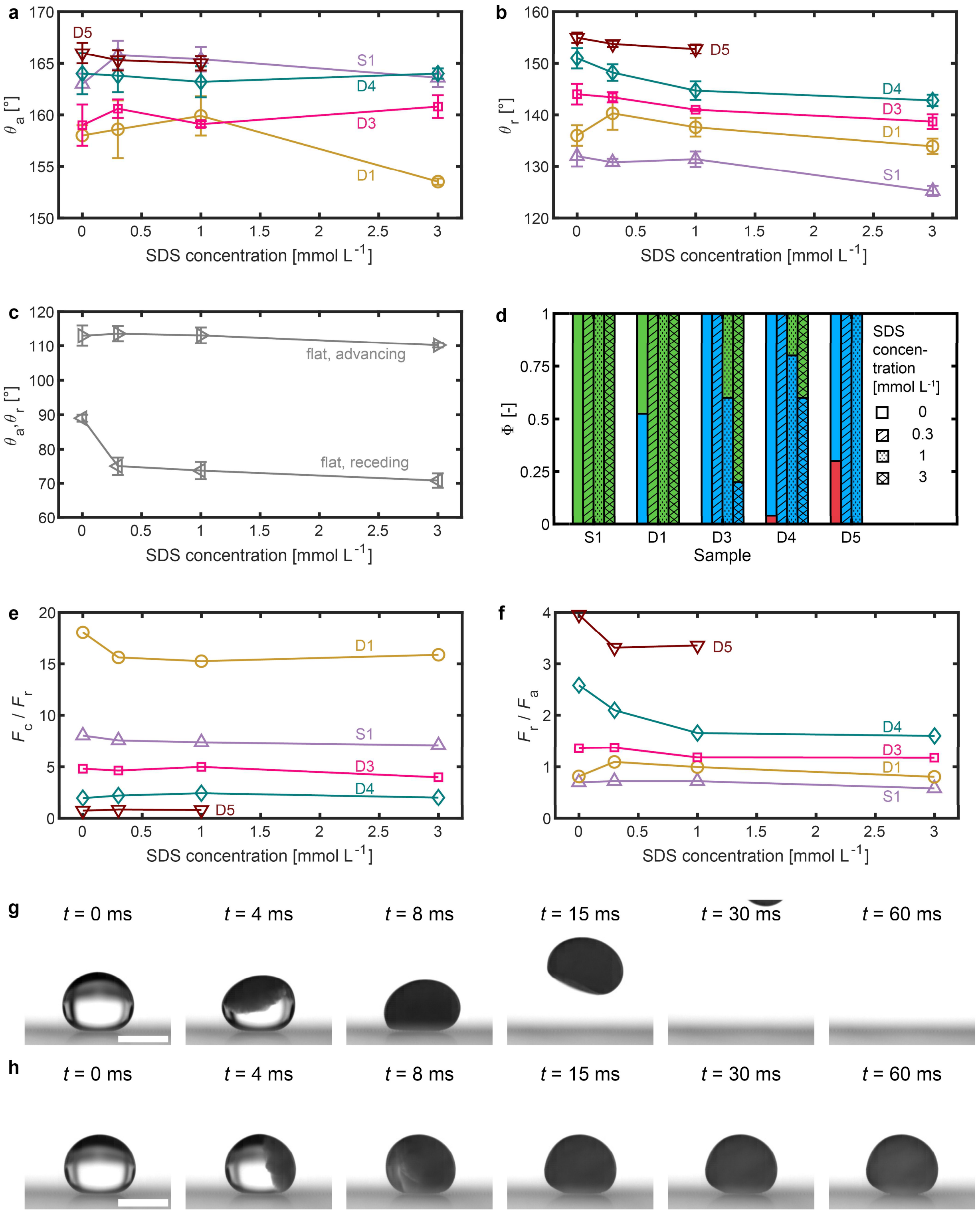


**Fig. S14. Effect of adding a surfactant to water on freezing-triggered wetting transition phenomena. a,b**, Advancing, $\theta_a$, and receding contact angles, $\theta_r$, respectively, measured for aqueous solutions of sodium dodecyl sulphate (SDS), as a function of SDS concentration and substrate (see Table S1 for details). Droplets with highest SDS concentration could not be placed

reliably in a Cassie state on D5 samples. Mean of n = 3 measurements per concentration and sample, error bars show standard deviation. **c**, $\theta_a$ and $\theta_r$, measured on flat (untextured) PDMS. Mean of n = 3 measurements per concentration and sample, error bars show standard deviation. **d**, Bar chart of outcome occurrence as a function of substrate and SDS concentration (plain bar, pure water, n ≥ 39, see Table S3 for details; hatched bar, 0.3 mmol $L^{-1}$; spotted bar, 1 mmol $L^{-1}$; crosshatched bar, 3 mmol $L^{-1}$; n = 10 for aqueous SDS solutions). Outcomes are differentiated by colour (red, impalement; blue, expulsion; green, suffusion). Whilst a pure water droplet always shows expulsion on a D3 substrate (suffusion occurrence 0%), adding 3 mmol $L^{-1}$ of SDS reduces the expulsion occurrence to only 20% and suffusion dominates (80%). Impalement was not observed for aqueous SDS solutions. **e,f**, Governing force ratios ($F_c$ / $F_r$) and ($F_r$ / $F_a$) plotted as a function of SDS concentration and substrate. Adding SDS to water reduces the surface tension, $\sigma$, while it also reduces $\theta_r$ and, hence, reduces the static contact angle, increasing the contact radius, $R_c$, of a droplet with a constant volume of 10 μL. Interestingly, the reductions in $\theta_r$ and the vapour pressure compensate for the reduction in $\sigma$, thereby reducing ($F_c$ / $F_r$) slightly for most samples when SDS is added. Moreover, ($F_r$ / $F_a$) is for most samples reduced by the addition of SDS, increasing the likelihood of suffusion. **g**, Image sequence of a droplet containing 0.3 mmol $L^{-1}$ of SDS undergoing expulsion on a D3 substrate. **h**, Image sequence of a droplet containing 3 mmol $L^{-1}$ of SDS undergoing suffusion on a D3 substrate. Initial droplet volume, $V$ = 10 μL. By adding SDS in different concentrations (0.3, 1, and 3 mmol $L^{-1}$), we have reduced the surface tension from the value of pure water at 0 °C ($\sigma$ = 75.64 mN $m^{-1}$) to values of 71.47, 68.49, and 57.28 mN $m^{-1}$, respectively.[20] For small amounts of SDS (as studied here), the saturated vapor pressure decreases with the addition of SDS.[17] Based on the literature data, we estimate the reduction in vapor pressure to be at least 0.6%, 2.1%, and 6.3% due to the addition of 0.3, 1, and 3 mmol $L^{-1}$, respectively.

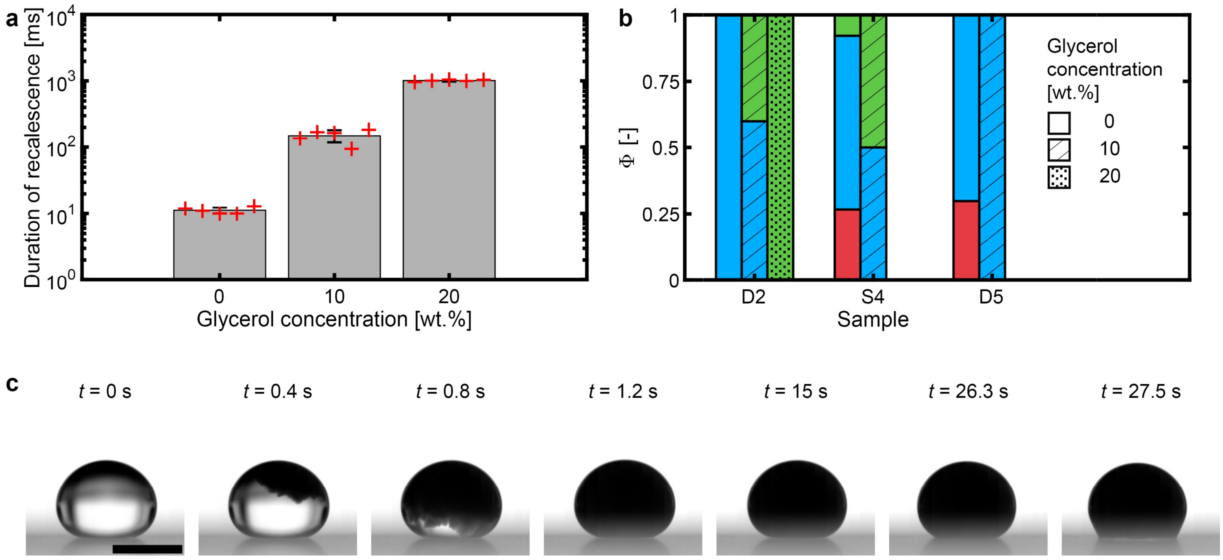


**Fig. S15. Effect of adding glycerol to water on freezing-triggered wetting transition phenomena. a**, Duration of recalescence (i.e., time for the freezing front of the first stage of freezing to traverse through the droplet) as a function of glycerol concentration in aqueous solutions, evaluated on a D2 sample, n = 5 per concentration, showing the mean ± standard deviation. **b**, Bar chart of outcome probability as a function of substrate and glycerol concentration (plain bar, pure water, n ≥ 40, see Table S3 for details; hatched bar, 10 wt.% glycerol; spotted bar, 20 wt.% glycerol). Outcomes are differentiated by colour (red, impalement; blue, expulsion; green, suffusion). Droplets with highest glycerol concentration could not be placed reliably in a Cassie state on S4 and D5 samples. **c**, Image sequence of a droplet containing 20 wt.% glycerol undergoing suffusion on a D2 substrate. Initial droplet volume, $V$ = 10 µL. Scale bar: 2 mm.

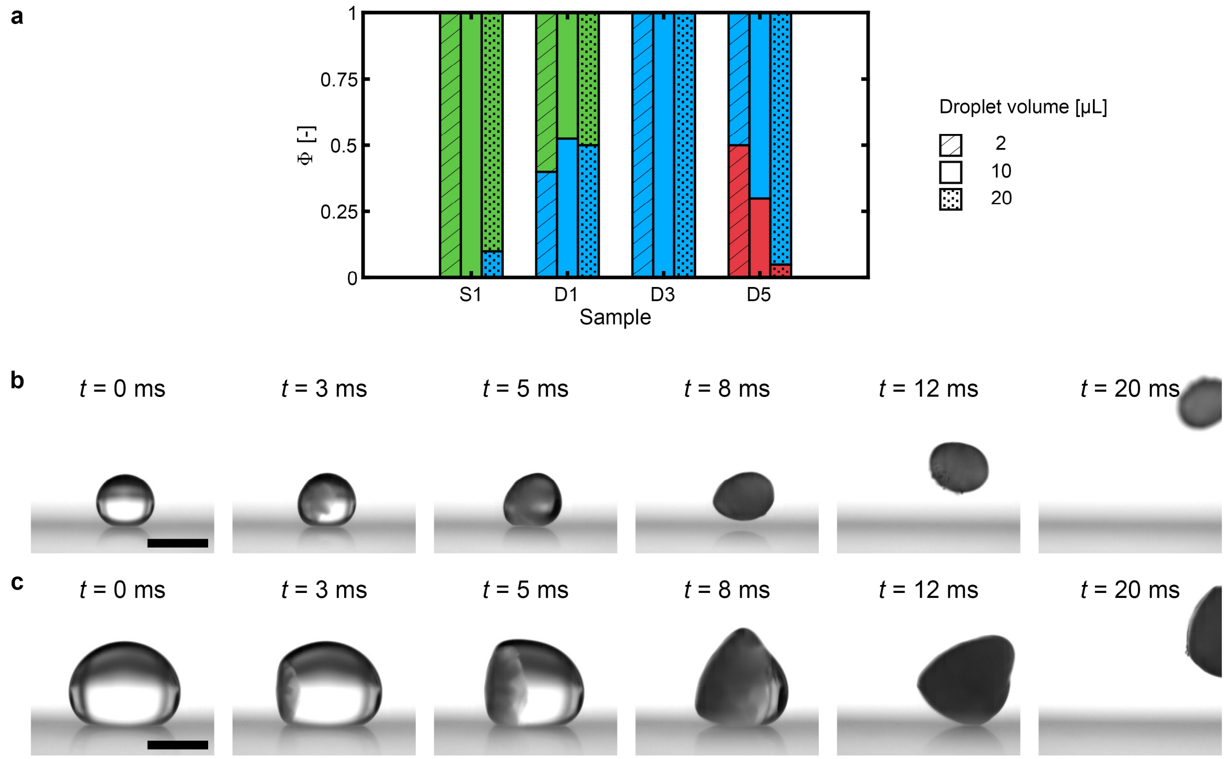


**Fig. S16. Effect of initial droplet volume on freezing-triggered wetting transition phenomena.** **a**, Experimental outcomes (differentiated by colour: red, impalement; blue, expulsion; green, suffusion) of droplet freezing events on PDMS micropillar substrates in a low-pressure environment as a function of the employed sample (S1, D1, D3, and D5 – see Table S1 for details) and the initial droplet volume (hatched bar, 2 µL, n = 10; plain bar, 10 µL, n ≥ 39; spotted bar, 20 µL, n ≥ 10; N = 249). For samples S1, D1 and D3, droplet volume has no major effect on the experimental outcome. For sample D5, increasing droplet volume increases the occurrence of expulsion. It was found that the large droplets on sample D5 nucleated relatively often from the lower hemisphere (nucleation angle larger than 90°), thereby reducing the impalement occurrence (see Fig. 2). **b**, Expulsion of a 2 µL droplet from a D3 sample. **c**, Expulsion of a 20 µL droplet from a D3 sample. (Scale bars: **b**, **c**, 2 mm.)

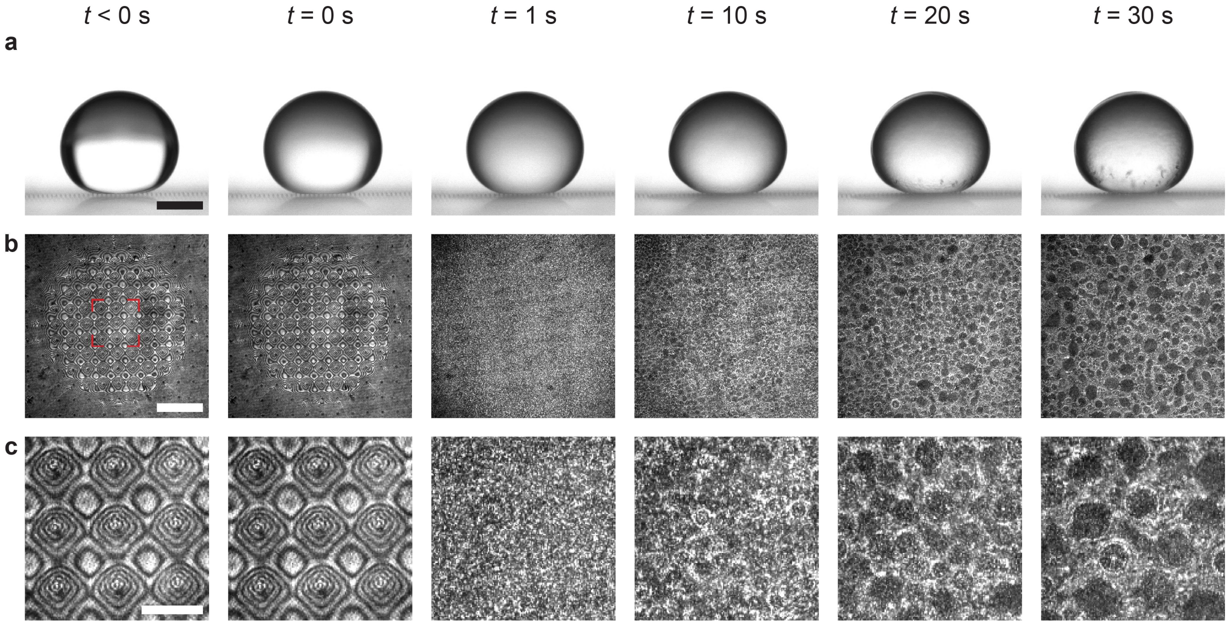


**Fig. S17. Freezing at ambient pressure with a wider pitch.** Synchronised (**a**) side- and (**b**) bottom-view image sequences of a water droplet freezing in a cold, dry environment at atmospheric pressure on a superhydrophobic PDMS texture [$d$, $s$, $h$] = [10, 100, 40] μm (sample D5). The wider pillar pitch results in larger and more amorphous frozen zones within the texture. (**c**) Enlarged frames of (**b**). (Scale bars: **a**, 1 mm; **b**, 300 μm; **c**, 100 μm)

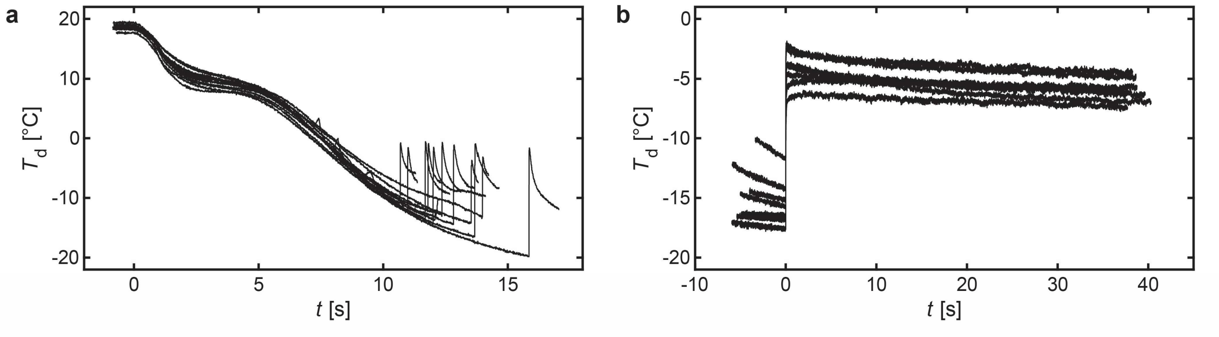


**Fig. S18. Temperature evolutions of droplets during freezing.** (**a**) Droplet temperature, $T_d$, *vs*. time, $t$, for low-pressure experiments with $t = 0$ s corresponding to the moment at which vacuum is pulled (N = 12). (**b**) $T_d$ *vs*. $t$ for ambient-pressure, reduced-temperature experiments with experiments synchronised to the moment of freezing nucleation (N = 7).

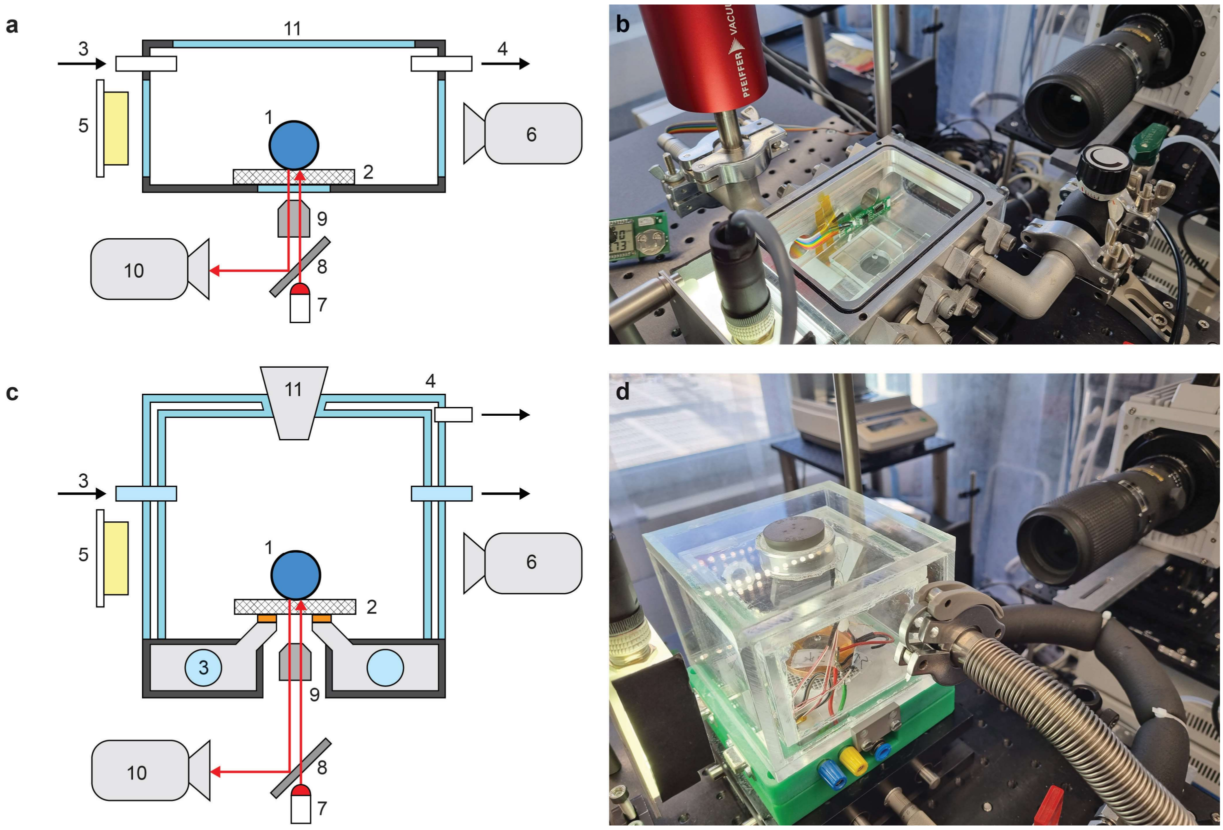


**Fig. S19. Experimental setups. a**, Schematic of the setup used for experiments in the low-pressure environment, composed of the droplet (1) placed on the substrate (2), gaseous nitrogen supply (3), connector to the vacuum pump (4), illumination (5) and high-speed camera for side-view visualisation (6), as well as illumination (7), beam splitter (8), objective (9) and high-speed camera (10) for bottom-view visualisation. The top plate (11) can be removed to access the chamber. **b**, Photograph of the setup used for experiments in the low-pressure environment. **c**, Schematic of the setup used for experiments in the ambient-pressure environment, composed of the droplet (1) placed on the substrate (2), cold gaseous nitrogen supply (3), double-walled insulation cover that can be evacuated (4), illumination (5) and high-speed camera for side-view visualisation (6), as well as illumination (7), beam splitter (8), objective (9) and high-speed camera (10) for bottom-view visualisation. The chamber can be opened from the top (11) to place droplets. **d**, Photograph of the setup used for experiments in the ambient-pressure environment.

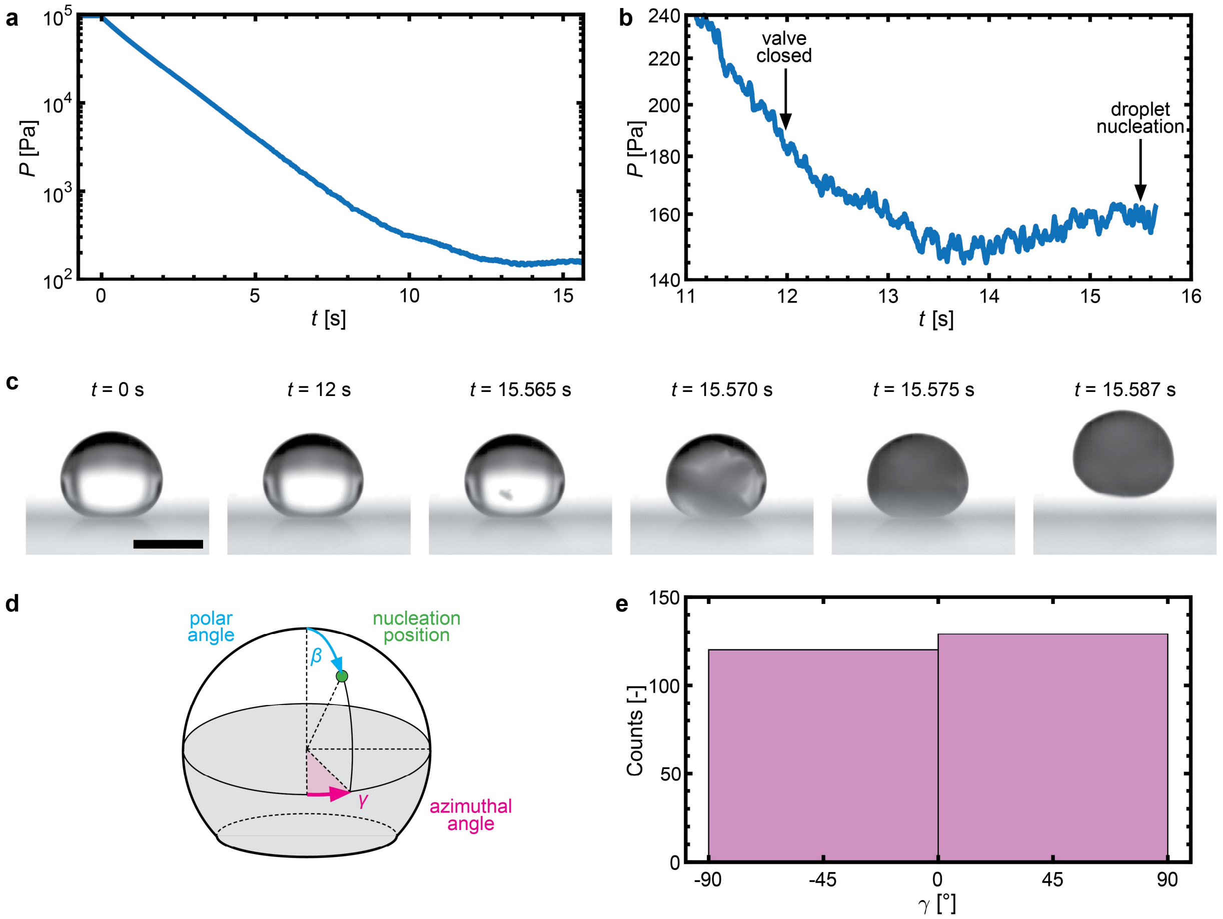


**Fig. S20. Excluding potential effect of convection on droplet nucleation. a**, Total chamber pressure, $P$, versus time, $t$, during an experiment with a modified experimental protocol. Whilst for regular low-pressure experiments the valve between the environmental chamber and the vacuum pump is open during the entire experiment, here, the valve was closed after approximately 12 seconds. By the time of droplet nucleation, the valve had already been closed for more than 3 seconds, thereby preventing any potential effect of convection related to chamber pump-down from affecting the droplet during freezing. **b**, Zoom-in on **a** showing how $P$ increases with $t$ at the moment of nucleation, which stems from droplet evaporation. **c**, Corresponding image sequence of a water droplet undergoing expulsion whilst the valve between environmental chamber and vacuum pump is closed. Initial droplet volume, $V = 10$ µL (see Movie S7). **d**, Definition of the azimuthal angle of nucleation, $\gamma$, where $\gamma = 0°$ represents the vertical centre line of the droplet from the side-view perspective. The outlet to the vacuum pump is on the left side of the droplet. A potential effect of convective cooling would be a bias in the azimuthal angle of nucleation (i.e., left or right from the observer's perspective).[8] Due to optical limitations from the side-view perspective, no $\gamma$ distinction is made between nucleation from the front or the rear of the droplet. **e**, Evaluation of the azimuthal angle for all 249 experiments shown in Fig. 2. 120 out of 249 droplets (48%) nucleated with $\gamma < 0°$, while the remaining 129 droplets (52%) nucleated with $\gamma \geq 0°$. No preferential azimuthal angle can be observed highlighting that convection is negligible compared to the substantial evaporative cooling of the droplet in the low-pressure environment (Scale bar: **c**, 2 mm.)

# Supplementary Tables

| Identifier | Material | Pitch ($s$) [µm] | Diameter ($d$) [µm] | Height ($h$) [µm] | Wetting Fraction ($f$) [-] | Capillary Pressure ($p_c$) [Pa] | Contact Angles ($\theta_a$ / $\theta_r$) [°] |
|---|---|---|---|---|---|---|---|
| D1 | PDMS | 33 | 10 | 25/40 | 0.072 | 919 | 158±1 / 136±2 |
| D2 | PDMS | 50 | 10 | 25/40 | 0.031 | 383 | 159±1 / 141±1 |
| D3 | PDMS | 56 | 10 | 25/40 | 0.025 | 304 | 159±2 / 144±2 |
| D4 | PDMS | 70 | 10 | 25/40 | 0.016 | 193 | 164±2 / 151±2 |
| D5 | PDMS | 100 | 10 | 25/40 | 0.008 | 94 | 166±1 / 155±1 |
| D6 | PDMS | 120 | 10 | 25/40 | 0.006 | 65 | 171±1 / 158±1 |
| S1 | PDMS | 100 | 42 | 29/48/83 | 0.139 | 453 | 163±1 / 132±2 |
| S2 | PDMS | 100 | 32 | 28/46/78 | 0.080 | 323 | 163±1 / 140±1 |
| S3 | PDMS | 100 | 22 | 27/43/73 | 0.038 | 212 | 165±1 / 148±1 |
| S4 | PDMS | 100 | 12 | 25/39/58 | 0.011 | 113 | 162±1 / 154±1 |
| C1 | Coating A on glass | 74 | 32 | 7 | 0.1469 | 1552 | 165±2 / 160±3 |
| C2 | Coating A on steel | 280 | 100 | - | 0.587 | 1624 | 164±2 / 155±3 |
| - | PDMS | - | - | - | - | - | 113±3 / 89±1 |
| D1* | Coating B on PDMS | 33 | 10 | 25 | 0.072 | 1748 | 167±2 / 143±2 |
| D6* | Coating B on PDMS | 120 | 10 | 25 | 0.006 | 123 | 170±2 / 160±2 |
| - | Coating B on PDMS | - | - | - | - | - | 138±3 / 86±2 |
| S1s | FDTS on silicon | 100 | 39 | 32 | 0.119 | 542 | 169±2 / 135±2 |
| S2s | FDTS on silicon | 100 | 30 | 31 | 0.071 | 395 | 169±1 / 142±1 |
| S3s | FDTS on silicon | 100 | 19 | 31 | 0.028 | 239 | 169±1 / 150±1 |
| S4s | FDTS on silicon | 100 | 9 | 31 | 0.006 | 111 | 170±1 / 159±2 |
| - | FDTS on silicon | - | - | - | - | - | 121±1 / 96±2 |

**Table S1. Details of substrates used in study.** Water contact angles are obtained by three independent measurements reporting the mean ± standard deviation. For surface C1, the height is given as the root-mean-square (RMS) as computed from the height profile (see SI, Capillary Pressure for details). The thermal conductivity in W $m^{-1}$ $K^{-1}$ of PDMS, glass, steel, and silicon are 0.16, 1, 15, and 150, respectively.[21] The elastic modulus in GPa of PDMS, glass, steel, and silicon are 0.003, 64, 190, and 149, respectively.[22]

| Parameter | Supercooled, low pressure | | Recalesced, low pressure | | Supercooled, ambient pressure | Recalesced, ambient pressure |
|---|---|---|---|---|---|---|
| $T_d$ [°C] | -15 | | 0 | | -20 | 0 |
| $P$ [Pa] | 72 | | 72 | | 96,000 | 96,000 |
| $T_{diffusion}$ [°C] | 0 | | 0 | | -20 | -10 |
| $\Delta x$ [mm] | 0.2 | | 0.2 | | 50 | 50 |
| $D_v$ [cm$^2$ s$^{-1}$] | 297 | | 297 | | 0.19 | 0.21 |
| $\xi_{kinetic}$ [m s$^{-1}$] | 1049 | | 1079 | | 1039 | 1079 |
| $\xi_{diffusion}$ [m s$^{-1}$] | 849 | | 849 | | $3.0 \cdot 10^8$ | $2.9 \cdot 10^8$ |
| $p_{v,surface}$ [Pa] | 191.3 | | 611.3 | | 125.5 | 611.3 |
| $RH$ [%] | 0 | 100 | 0 | 100 | 0 | 0 |
| $p_{v,\infty}$ [Pa] | 0 | 72 | 0 | 72 | 0 | 0 |
| $j$ [kg m$^{-2}$ s$^{-1}$] | 0.101 | 0.063 | 0.317 | 0.280 | $4.2 \cdot 10^{-7}$ | $2.1 \cdot 10^{-6}$ |

**Table S2. Vapour flux modelling.** Assumptions, measurements and results of the vapour flux modelling.

| Identifier | $F_c / F_r$ | $F_r / F_a$ | Number of experiments (n) | Impalement occurrence [%] | Suffusion occurrence [%] | Expulsion occurrence [%] |
|---|---|---|---|---|---|---|
| D1 | 18.1 | 0.8 | 60 | 0 | 50 | 50 |
| D2 | 6.6 | 1.1 | 40 | 0 | 0 | 100 |
| D3 | 4.8 | 1.4 | 60 | 0 | 0 | 100 |
| D4 | 2.0 | 2.6 | 50 | 4 | 0 | 96 |
| D5 | 0.7 | 4.0 | 60 | 28 | 0 | 72 |
| D6 | 0.3 | 6.6 | 39 | 54 | 0 | 46 |
| S1 | 8.0 | 0.7 | 59 | 0 | 98 | 2 |
| S2 | 4.8 | 1.1 | 72 | 0 | 79 | 21 |
| S3 | 2.3 | 2.0 | 81 | 0 | 41 | 59 |
| S4 | 1.1 | 3.3 | 90 | 27 | 8 | 66 |
| C1 | 6.7 | 6.8 | 10 | 0 | 0 | 100 |
| C2 | 5.9 | 3.8 | 10 | 0 | 0 | 100 |
| D1* | 20.7 | 1.5 | 10 | 0 | 0 | 100 |
| D6* | 0.6 | 7.9 | 10 | 0 | 0 | 100 |

**Table S3. Details of results plotted in Fig. 3.**

Supplementary Videos

**Video S1: Impalement of a supercooled droplet during freezing on a superhydrophobic substrate in a low-pressure environment.**

**Video S2: Rapid expulsion of a supercooled droplet during freezing on a superhydrophobic surface in a low-pressure environment.**

**Video S3: Top-down suffusion of a post-recalescence droplet into a superhydrophobic surface in a low-pressure environment.**

**Video S4: Expulsion event on a porous superhydrophobic mesh in a low-pressure environment.**

**Video S5: Expulsion event on an HFS-modified hierarchical superhydrophobic surface in a low-pressure environment.**

**Video S6: Bottom-up suffusion of ice slush within a superhydrophobic texture beneath a post-recalescence droplet cooled in an atmospheric-pressure, low-temperature environment.**

**Video S7: Expulsion event whilst the valve between environmental chamber and vacuum pump is closed, thereby excluding a potential effect of convection on droplet nucleation.**
